# Supplementary material for: Systematic Studies of the Circadian Clock Genes Impact on Temperature Compensation and Cell Proliferation Using CRISPR Tools
Source: Biology (Basel). 2021 Nov 18;10(11):1204. doi: 10.3390/biology10111204 (PMC8614980; doi:10.3390/biology10111204)
Supplement: Supplementary file 1 [file biology-10-01204-s001.zip › biology-1433404-supplementary.pdf]

Supplemental Materials

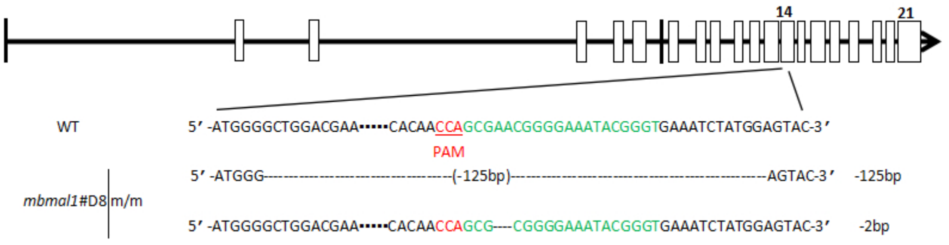

Figure S1. The sgRNA targeting the *Bmal1* gene and the sequences of both alleles in the *Bmal1* deletion cell strain.

```
mbmal1      MADQRMDISS TISDFMSPGP TDLLSGSLGT SGVDCNRKRK GSATDYQESM DTDKDDPHGR
D8-2        MADQRMDISS TISDFMSPGP TDLLSGSLGT SGVDCNRKRK GSATDYQESM DTDKDDPHGR
D8-1        MADQRMDISS TISDFMSPGP TDLLSGSLGT SGVDCNRKRK GSATDYQESM DTDKDDPHGR
            ********** ********** ********** ********** ********** **********

mbmal1      LEYAEHQGRI KNAREAHSQI EKRRRDKMNS FIDELASLVP TCNAMSRKLD KLTVLRMAVQ
D8-2        LEYAEHQGRI KNAREAHSQI EKRRRDKMNS FIDELASLVP TCNAMSRKLD KLTVLRMAVQ
D8-1        LEYAEHQGRI KNAREAHSQI EKRRRDKMNS FIDELASLVP TCNAMSRKLD KLTVLRMAVQ
            ********** ********** ********** ********** ********** **********

mbmal1      HMKTLRGATN PYTEANYKPT FLSDDELKHL ILRAADGFLF VVGCDRGKIL FVSESVFKIL
D8-2        HMKTLRGATN PYTEANYKPT FLSDDELKHL ILRAADGFLF VVGCDRGKIL FVSESVFKIL
D8-1        HMKTLRGATN PYTEANYKPT FLSDDELKHL ILRAADGFLF VVGCDRGKIL FVSESVFKIL
            ********** ********************* ********** ********** **********

mbmal1      NYSQNDLIGQ SLFDYLHPKD IAKVKEQLSS SDTAPRERLI DAKTGLPVKT DITPGPSRLC
D8-2        NYSQNDLIGQ SLFDYLHPKD IAKVKEQLSS SDTAPRERLI DAKTGLPVKT DITPGPSRLC
D8-1        NYSQNDLIGQ SLFDYLHPKD IAKVKEQLSS SDTAPRERLI DAKTGLPVKT DITPGPSRLC
            ********** ********** ********** ********************* **********

mbmal1      SGARRSFFCR MKCNRPSVKV EDKDFASTCS KKKADRKSFC TIHSTGYLKS WPPTKMGLDE
D8-2        SGARRSFFCR MKCNRPSVKV EDKDFASTCS KKKADRKSFC TIHSTGYLKS WPPTKMGVRF
D8-1        SGARRSFFCR MKCNRPSVKV EDKDFASTCS KKKADRKSFC TIHSTGYLKS WPPTKMGLDE
            ********** ********** ********** ********** ********** *******

mbmal1      DNEPDNEGCN LSCLVAIGRL HSHMVPQPAN GEIRVKSMEY VSRHAIDGKF VFVDQRATAI
D8-2        STRNRWEICF CRSEGDSYFG VSTTGTSRYI ML*------- ---------- ----------
D8-1        DNEPDNEGCN LSCLVAIGRL HSHMVPQPAR GNTGEIYGVR FSTRNRWEIC FCRSEGDSYF

mbmal1      LAYLPQELLG TSCYEYFHQD DIGHLAECHR QVLQTREKIT TNCYKFKIKD GSFITLRSRW
D8-2        ---------- ---------- ---------- ---------- ---------- ----------
D8-1        GVSTTGTSRY IML*------ ---------- ---------- ---------- ----------

mbmal1      FSFMNPWTKE VEYIVSTNTV VLANVLEGGD PTFPQLTAPP HSMDSMLPSG EGGPKRTHPT
D8-2        ---------- ---------- ---------- ---------- ---------- ----------
D8-1        ---------- ---------- ---------- ---------- ---------- ----------

mbmal1      VPGIPGGTRA GAGKIGRMIA EEIMEIHRIR GSSPSSCGSS PLNITSTPPP DASSPGGKKI
D8-2        ---------- ---------- ---------- ---------- ---------- ----------
D8-1        ---------- ---------- ---------- ---------- ---------- ----------

mbmal1      LNGGTPDIPS TGLLPGQAQE TPGYPYSDSS SILGENPHIG IDMIDNDQGS SSPSNDEAAM
D8-2        ---------- ---------- ---------- ---------- ---------- ----------
D8-1        ---------- ---------- ---------- ---------- ---------- ----------

mbmal1      AVIMSLLEAD AGLGGPVDFS DLPWPL*
D8-2        ---------- ---------- -------
D8-1        ---------- ---------- -------
```

Figure S2. Predicted translation products of both alleles in the *Bmal1* deletion cell strain.

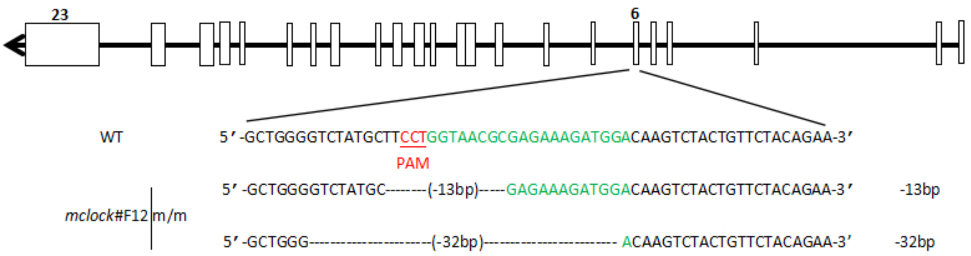

Figure S3. The sgRNA targeting the *Clock* gene and the sequences of both alleles in the *Clock* deletion cell strain.

```
mclock    MVFTVSCSKM SSIVDRDDSS IFDGLVEEDD KDKAKRVSRN KSEKKRRDQF NVLIKELGSM
F12-1     MVFTVSCSKM SSIVDRDDSS IFDGLVEEDD KDKAKRVSRN KSEKKRRDQF NVLIKELGSM
F12-2     MVFTVSCSKM SSIVDRDDSS IFDGLVEEDD KDKAKRVSRN KSEKKRRDQF NVLIKELGQV
          ********** ********** ********** ********** ********** ********

mclock    LPGNARKMDK STVLQKSIDF LRKHKETTAQ SDASEIRQDW KPTFLSNEEF TQLMLEALDG
F12-1     RERWTSLLFY RRALIFCANI KRPLHSQMLV RFDRTGNPHS LVMKSLHS*- ----------
F12-2     YCSTEEH*-- ---------- ---------- ---------- ---------- ----------

mclock    FFLAIMTDGS IIYVSESVTS LLEHLPSDLV DQSIFNFIPE GEHSEVYKIL STHLLESDSL
F12-1     ---------- ---------- ---------- ---------- ---------- ----------
F12-2     ---------- ---------- ---------- ---------- ---------- ----------

mclock    TPEYLKSKNQ LEFCCHMLRG TIDPKEPSTY EYVRFIGNFK SLTSVSTSTH NGFEGTIQRT
F12-1     ---------- ---------- ---------- ---------- ---------- ----------
F12-2     ---------- ---------- ---------- ---------- ---------- ----------

mclock    HRPSYEDRVC FVATVRLATP QFIKEMCTVE EPNEEFTSRH SLEWKFLFLD HRAPPIIGYL
F12-1     ---------- ---------- ---------- ---------- ---------- ----------
F12-2     ---------- ---------- ---------- ---------- ---------- ----------

mclock    PFEVLGTSGY DYYHVDDLEN LAKCHEHLMQ YGKGKSCYYR FLTKGQQWIW LQTHYYITYH
F12-1     ---------- ---------- ---------- ---------- ---------- ----------
F12-2     ---------- ---------- ---------- ---------- ---------- ----------

mclock    QWNSRPEFIV CTHTVVSYAE VRAERRRELG IEESLPETAA DKSQDSGSDN RINTVSLKEA
F12-1     ---------- ---------- ---------- ---------- ---------- ----------
F12-2     ---------- ---------- ---------- ---------- ---------- ----------

mclock    LERFDHSPTP SASSRSSRKS SHTAVSDPSS TPTKIPTDTS TPPRQHLPAH EKMTQRRSSF
F12-1     ---------- ---------- ---------- ---------- ---------- ----------
F12-2     ---------- ---------- ---------- ---------- ---------- ----------

mclock    SSQSINSQSV GPSLTQPAMS QAANLPIPQG MSQFQFSAQL GAMQHLKDQL EQRTRMIEAN
F12-1     ---------- ---------- ---------- ---------- ---------- ----------
F12-2     ---------- ---------- ---------- ---------- ---------- ----------

mclock    IHRQQEELRK IQEQLQMVHG QGLQMFLQQS NPGLNFGSVQ LSSGNSNIQQ LTPVNMQGQV
F12-1     ---------- ---------- ---------- ---------- ---------- ----------
F12-2     ---------- ---------- ---------- ---------- ---------- ----------

mclock    VPANQVQSGH ISTGQHMIQQ QTLQSTSTQQ SQQSVMSGHS QQTSLPSQTP STLTAPLYNT
F12-1     ---------- ---------- ---------- ---------- ---------- ----------
F12-2     ---------- ---------- ---------- ---------- ---------- ----------

mclock    MVISQPAAGS MVQIPSSMPQ NSTQSATVTT FTQDRQIRFS QGQQLVTKLV TAPVACGAVM
F12-1     ---------- ---------- ---------- ---------- ---------- ----------
F12-2     ---------- ---------- ---------- ---------- ---------- ----------

mclock    VPSTMLMGQV VTAYPTFATQ QQQAQTLSVT QQQQQQQQQP PQQQQQQQQS SQEQQLPSVQ
F12-1     ---------- ---------- ---------- ---------- ---------- ----------
F12-2     ---------- ---------- ---------- ---------- ---------- ----------

mclock    QPAQAQLGQP PQQFLQTSRL LHGNPSTQLI LSAAFPLQQS TFPPSHHQQH QPQQQQQLPR
F12-1     ---------- ---------- ---------- ---------- ---------- ----------
F12-2     ---------- ---------- ---------- ---------- ---------- ----------

mclock    HRTDSLTDPS KVQPQ*
F12-1     ---------- ------
F12-2     ---------- ------
```

Figure S4. Predicted translation products of both alleles in the *Clock* deletion cell strain.

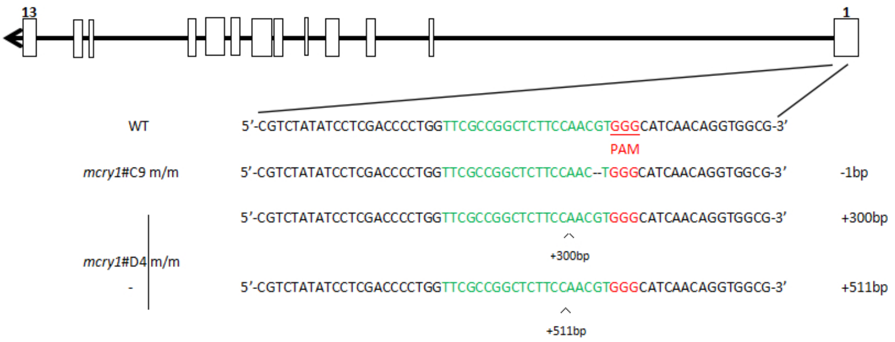

Figure S5. The sgRNA targeting the *Cry1* gene and the sequences of both alleles in the *Cry1* deletion cell strains.

```
mcry1     MGVNAVHWFR KGLRLHDNPA LKECIQGADT IRCVYILDPW FAGSSNVGIN RWRFLLQCLE
C9-1/2    MGVNAVHWFR KGLRLHDNPA LKECIQGADT IRCVYILDPW FAGSSNWAST GGDFCFSVLR
          ********** ********** ********** ********** ******

mcry1     DLDANLRKLN SRLFVIRGQP ADVFPRLFKE WNITKLSIEY DSEPFGKERD AAIKKLATEA
C9-1/2    ILMPIYEN*- ---------- ---------- ---------- ---------- ----------

mcry1     GVEVIVRISH TLYDLDKIIE LNGGQPPLTY KRFQTLVSKM EPLEMPADTI TSDVIGKCMT
C9-1/2    ---------- ---------- ---------- ---------- ---------- ----------

mcry1     PLSDDHDEKY GVPSLEELGF DTDGLSSAVW PGGETEALTR LERHLERKAW VANFERPRMN
C9-1/2    ---------- ---------- ---------- ---------- ---------- ----------

mcry1     ANSLLASPTG LSPYLRFGCL SCRLFYFKLT DLYKKVKKNS SPPLSLYGQL LWREFFYTAA
C9-1/2    ---------- ---------- ---------- ---------- ---------- ----------

mcry1     TNNPRFDKME GNPICVQIPW DKNPEALAKW AEGRTGFPWI DAIMTQLRQE GWIHHLARHA
C9-1/2    ---------- ---------- ---------- ---------- ---------- ----------

mcry1     VACFLTRGDL WISWEEGMKV FEELLLDADW SINAGSWMWL SCSSFFQQFF HCYCPVGFGR
C9-1/2    ---------- ---------- ---------- ---------- ---------- ----------

mcry1     RTDPNGDYIR RYLPVLRGFP AKYIYDPWNA PEGIQKVAKC LIGVNYPKPM VNHAEASRLN
C9-1/2    ---------- ---------- ---------- ---------- ---------- ----------

mcry1     IERMKQIYQQ LSRYRGLGLL ASVPSNSNGN GGLMGYAPGE NVPSCSSSGN GGLMGYAPGE
C9-1/2    ---------- ---------- ---------- ---------- ---------- ----------

mcry1     NVPSCSGGNC SQGSGILHYA HGDSQQTHSL KQGRSSAGTG LSSGKRPSQE EDAQSVGPKV
C9-1/2    ---------- ---------- ---------- ---------- ---------- ----------

mcry1     QRQSSN*
C9-1/2    -------
```

Figure S6. Predicted translation products of both alleles in the *Cry1* deletion cell strain (*Cry1*-C9).

```
mcry1   MGVNAVHWFR KGLRLHDNPA LKECIQGADT IRCVYILDPW FAGSSNVGIN RWRFLLQCLE
D4-1    MGVNAVHWFR KGLRLHDNPA LKECIQGADT IRCVYILDPW FAGSSKIRFV FIEHRQTRSG
D4-2    MGVNAVHWFR KGLRLHDNPA LKECIQGADT IRCVYILDPW FAGSSNSRVT TGNSGCLLCW
        ********** ********** ********** ********** *****

mcry1   DLDANLRKLN SRLFVIRGQP ADVFPRLFKE WNITKLSIEY DSEPFGKERD AAIKKLATEA
D4-1    S*-------- ---------- ---------- ---------- ---------- ----------
D4-2    PRQVQSSIRV AKESWGLLSS DCRANRPHLG LCPEASVPLQ GRHPRGRLWQ CAHPQPLSPL

mcry1   GVEVIVRISH TLYDLDKIIE LNGGQPPLTY KRFQTLVSKM EPLEMPADTI TSDVIGKCMT
D4-1    ---------- ---------- ---------- ---------- ---------- ----------
D4-2    QGRLAPRSEG KARAELRHQS GTTHGSHTPA ACSFPGANAR SWAGLGEARP AQSSLSAGAL

mcry1   PLSDDHDEKY GVPSLEELGF DTDGLSSAVW PGGETEALTR LERHLERKAW VANFERPRMN
D4-1    ---------- ---------- ---------- ---------- ---------- ----------
D4-2    CYRAWAQSWS SAGVEAHSVL PSTGLQAPAG SQPLLSRGHQ QVAIFASVS* ----------

mcry1   ANSLLASPTG LSPYLRFGCL SCRLFYFKLT DLYKKVKKNS SPPLSLYGQL LWREFFYTAA
D4-1    ---------- ---------- ---------- ---------- ---------- ----------
D4-2    ---------- ---------- ---------- ---------- ---------- ----------

mcry1   TNNPRFDKME GNPICVQIPW DKNPEALAKW AEGRTGFPWI DAIMTQLRQE GWIHHLARHA
D4-1    ---------- ---------- ---------- ---------- ---------- ----------
D4-2    ---------- ---------- ---------- ---------- ---------- ----------

mcry1   VACFLTRGDL WISWEEGMKV FEELLLDADW SINAGSWMWL SCSSFFQQFF HCYCPVGFGR
D4-1    ---------- ---------- ---------- ---------- ---------- ----------
D4-2    ---------- ---------- ---------- ---------- ---------- ----------

mcry1   RTDPNGDYIR RYLPVLRGFP AKYIYDPWNA PEGIQKVAKC LIGVNYPKPM VNHAEASRLN
D4-1    ---------- ---------- ---------- ---------- ---------- ----------
D4-2    ---------- ---------- ---------- ---------- ---------- ----------

mcry1   IERMKQIYQQ LSRYRGLGLL ASVPSNSNGN GGLMGYAPGE NVPSCSSSGN GGLMGYAPGE
D4-1    ---------- ---------- ---------- ---------- ---------- ----------
D4-2    ---------- ---------- ---------- ---------- ---------- ----------

mcry1   NVPSCSGGNC SQGSGILHYA HGDSQQTHSL KQGRSSAGTG LSSGKRPSQE EDAQSVGPKV
D4-1    ---------- ---------- ---------- ---------- ---------- ----------
D4-2    ---------- ---------- ---------- ---------- ---------- ----------

mcry1   QRQSSN*
D4-1    -------
D4-2    -------
```

Figure S7. Predicted translation products of both alleles in the *Cry1* deletion cell strain (*Cry1*-D4).

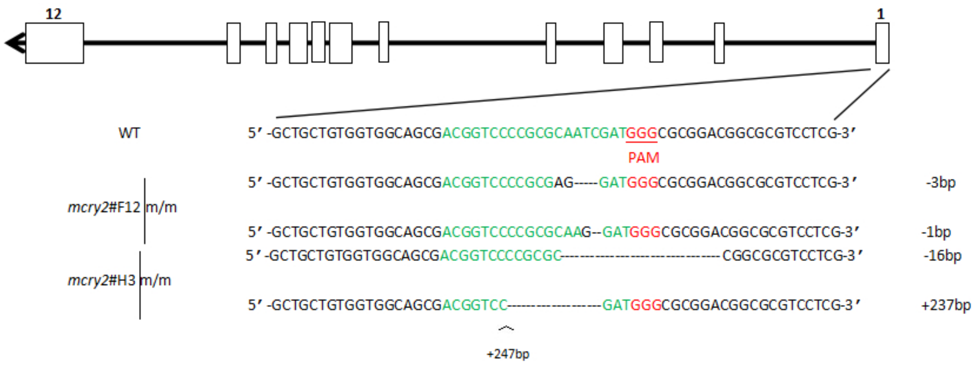

Figure S8. The sgRNA targeting the *Cry2* gene and the sequences of both alleles in the *Cry2* deletion cell strain.

```
mcry2   MAAAAVVAAT VPAQSMGADG ASSVHWFRKG LRLHDNPALL AAVRGARCVR CVYILDPWFA
F12-1   MAAAAVVAAT VPAQGWARTA RPRCTGSAKD YGSTTTPRC* ---------- ----------
F12-2   MAAAAVVAAT VPAR-MGADG ASSVHWFRKG LRLHDNPALL AAVRGARCVR CVYILDPWFA
        ********** ***

mcry2   ASSSVGINRW RFLLQSLEDL DTSLRKLNSR LFVVRGQPAD VFPRLFKEWG VTRLTFEYDS
F12-1   ---------- ---------- ---------- ---------- ---------- ----------
F12-2   ASSSVGINRW RFLLQSLEDL DTSLRKLNSR LFVVRGQPAD VFPRLFKEWG VTRLTFEYDS

mcry2   EPFGKERDAA IMKMAKEAGV EVVTENSHTL YDLDRIIELN GQKPPLTYKR FQALISRMEL
F12-1   ---------- ---------- ---------- ---------- ---------- ----------
F12-2   EPFGKERDAA IMKMAKEAGV EVVTENSHTL YDLDRIIELN GQKPPLTYKR FQALISRMEL

mcry2   PKKPAVAVSS QQMESCRAEI QENHDDTYGV PSLEELGFPT EGLGPAVWQG GETEALARLD
F12-1   ---------- ---------- ---------- ---------- ---------- ----------
F12-2   PKKPAVAVSS QQMESCRAEI QENHDDTYGV PSLEELGFPT EGLGPAVWQG GETEALARLD

mcry2   KHLERKAWVA NYERPRMNAN SLLASPTGLS PYLRFGCLSC RLFYYRLWDL YKKVKRNSTP
F12-1   ---------- ---------- ---------- ---------- ---------- ----------
F12-2   KHLERKAWVA NYERPRMNAN SLLASPTGLS PYLRFGCLSC RLFYYRLWDL YKKVKRNSTP

mcry2   PLSLFGQLLW REFFYTAATN NPRFDRMEGN PICIQIPWDR NPEALAKWAE GKTGFPWIDA
F12-1   ---------- ---------- ---------- ---------- ---------- ----------
F12-2   PLSLFGQLLW REFFYTAATN NPRFDRMEGN PICIQIPWDR NPEALAKWAE GKTGFPWIDA

mcry2   IMTQLRQEGW IHHLARHAVA CFLTRGDLWV SWESGVRVFD ELLLDADFSV NAGSWMWLSC
F12-1   ---------- ---------- ---------- ---------- ---------- ----------
F12-2   IMTQLRQEGW IHHLARHAVA CFLTRGDLWV SWESGVRVFD ELLLDADFSV NAGSWMWLSC

mcry2   SAFFQQFFHC YCPVGFGRRT DPSGDYIRRY LPKLKGFPSR YIYEPWNAPE SVQKAAKCII
F12-1   ---------- ---------- ---------- ---------- ---------- ----------
F12-2   SAFFQQFFHC YCPVGFGRRT DPSGDYIRRY LPKLKGFPSR YIYEPWNAPE SVQKAAKCII

mcry2   GVDYPRPIVN HAETSRLNIE RMKQIYQQLS RYRGLCLLAS VPSCVEDLSH PVAEPGSSQA
F12-1   ---------- ---------- ---------- ---------- ---------- ----------
F12-2   GVDYPRPIVN HAETSRLNIE RMKQIYQQLS RYRGLCLLAS VPSCVEDLSH PVAEPGSSQA

mcry2   GSISNTGPRA LSSGPASPKR KLEAAEEPPG EELTKRARVT EMPTQEPASK DS*
F12-1   ---------- ---------- ---------- ---------- ---------- ---
F12-2   GSISNTGPRA LSSGPASPKR KLEAAEEPPG EELTKRARVT EMPTQEPASK DS*
```

Figure S9. Predicted translation products of both alleles in the *Cry2* deletion cell strain (*Cry2*-F12).

```
mcry2      MAAAAVVAAT VPAQSMGADG ASSVHWFRKG LRLHDNPALL AAVRGARCVR CVYILDPWFA
H3-1       MAAAAVVAAT VRVLHRV*-- ---------- ---------- ---------- ----------
H3-2       MAAAAVVAAT VPAPARPRCT GSAKDYGSTT TPRC*----- ---------- ----------
           ********** *
mcry2      ASSSVGINRW RFLLQSLEDL DTSLRKLNSR LFVVRGQPAD VFPRLFKEWG VTRLTFEYDS
H3-1       ---------- ---------- ---------- ---------- ---------- ----------
H3-2       ---------- ---------- ---------- ---------- ---------- ----------

mcry2      EPFGKERDAA IMKMAKEAGV EVVTENSHTL YDLDRIIELN GQKPPLTYKR FQALISRMEL
H3-1       ---------- ---------- ---------- ---------- ---------- ----------
H3-2       ---------- ---------- ---------- ---------- ---------- ----------

mcry2      PKKPAVAVSS QQMESCRAEI QENHDDTYGV PSLEELGFPT EGLGPAVWQG GETEALARLD
H3-1       ---------- ---------- ---------- ---------- ---------- ----------
H3-2       ---------- ---------- ---------- ---------- ---------- ----------

mcry2      KHLERKAWVA NYERPRMNAN SLLASPTGLS PYLRFGCLSC RLFYYRLWDL YKKVKRNSTP
H3-1       ---------- ---------- ---------- ---------- ---------- ----------
H3-2       ---------- ---------- ---------- ---------- ---------- ----------

mcry2      PLSLFGQLLW REFFYTAATN NPRFDRMEGN PICIQIPWDR NPEALAKWAE GKTGFPWIDA
H3-1       ---------- ---------- ---------- ---------- ---------- ----------
H3-2       ---------- ---------- ---------- ---------- ---------- ----------

mcry2      IMTQLRQEGW IHHLARHAVA CFLTRGDLWV SWESGVRVFD ELLLDADFSV NAGSWMWLSC
H3-1       ---------- ---------- ---------- ---------- ---------- ----------
H3-2       ---------- ---------- ---------- ---------- ---------- ----------

mcry2      SAFFQQFFHC YCPVGFGRRT DPSGDYIRRY LPKLKGFPSR YIYEPWNAPE SVQKAAKCII
H3-1       ---------- ---------- ---------- ---------- ---------- ----------
H3-2       ---------- ---------- ---------- ---------- ---------- ----------

mcry2      GVDYPRPIVN HAETSRLNIE RMKQIYQQLS RYRGLCLLAS VPSCVEDLSH PVAEPGSSQA
H3-1       ---------- ---------- ---------- ---------- ---------- ----------
H3-2       ---------- ---------- ---------- ---------- ---------- ----------

mcry2      GSISNTGPRA LSSGPASPKR KLEAAEEPPG EELTKRARVT EMPTQEPASK DS*
H3-1       ---------- ---------- ---------- ---------- ---------- ---
H3-2       ---------- ---------- ---------- ---------- ---------- ---
```

Figure S10. Predicted translation products of both alleles in the *Cry2* deletion cell strain (*Cry2*-H3).

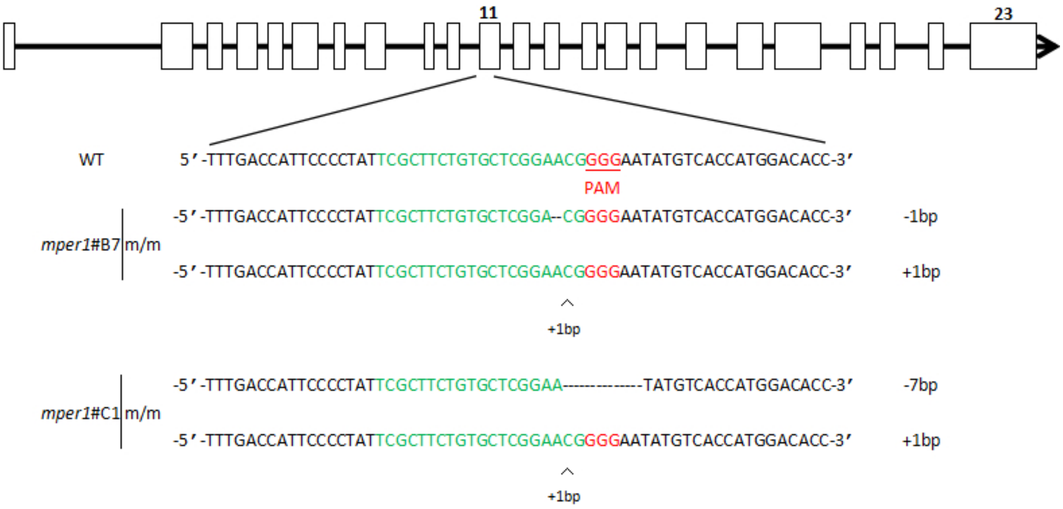

Figure S11. The sgRNA targeting the *Per1* gene and the sequences of both alleles in the *Per1* deletion cell strain.

```
mper1       MSGPLEGADG GGDPRPGEPF CPGGVPSPGA PQHRPCPGPS LADDTDANSN GSSGNESNGP
C1-1        MSGPLEGADG GGDPRPGEPF CPGGVPSPGA PQHRPCPGPS LADDTDANSN GSSGNESNGP
C1-2        MSGPLEGADG GGDPRPGEPF CPGGVPSPGA PQHRPCPGPS LADDTDANSN GSSGNESNGP
            ********** ********** ********** ********** ********** **********
mper1       ESRGASQRSS HSSSSGNGKD SALLETTESS KSTNSQSPSP PSSSIAYSLL SASSEQDNPS
C1-1        ESRGASQRSS HSSSSGNGKD SALLETTESS KSTNSQSPSP PSSSIAYSLL SASSEQDNPS
C1-2        ESRGASQRSS HSSSSGNGKD SALLETTESS KSTNSQSPSP PSSSIAYSLL SASSEQDNPS
            ********** ********** ********** ********** ********** **********
mper1       TSGCSSEQSA RARTQKELMT ALRELKLRLP PERRGKGRSG TLATLQYALA CVKQVQANQE
C1-1        TSGCSSEQSA RARTQKELMT ALRELKLRLP PERRGKGRSG TLATLQYALA CVKQVQANQE
C1-2        TSGCSSEQSA RARTQKELMT ALRELKLRLP PERRGKGRSG TLATLQYALA CVKQVQANQE
            ********** ********** ********** ********** ********** **********
mper1       YYQQWSLEEG EPCAMDMSTY TLEELEHITS EYTLRNQDTF SVAVSFLTGR IVYISEQAGV
C1-1        YYQQWSLEEG EPCAMDMSTY TLEELEHITS EYTLRNQDTF SVAVSFLTGR IVYISEQAGV
C1-2        YYQQWSLEEG EPCAMDMSTY TLEELEHITS EYTLRNQDTF SVAVSFLTGR IVYISEQAGV
            ********** ********** ********** ********** ********** **********
mper1       LLRCKRDVFR GARFSELLAP QDVGVFYGST TPSRLPTWGT GTSAGSGLKD FTQEKSVFCR
C1-1        LLRCKRDVFR GARFSELLAP QDVGVFYGST TPSRLPTWGT GTSAGSGLKD FTQEKSVFCR
C1-2        LLRCKRDVFR GARFSELLAP QDVGVFYGST TPSRLPTWGT GTSAGSGLKD FTQEKSVFCR
            ********** ********** ********** ********** ********** **********
mper1       IRGGPDRDPG PRYQPFRLTP YVTKIRVSDG APAQPCCLLI AERIHSGYEA PRIPPDKRIF
C1-1        IRGGPDRDPG PRYQPFRLTP YVTKIRVSDG APAQPCCLLI AERIHSGYEA PRIPPDKRIF
C1-2        IRGGPDRDPG PRYQPFRLTP YVTKIRVSDG APAQPCCLLI AERIHSGYEA PRIPPDKRIF
            ********** ********** ********** ********** ********** **********
mper1       TTRHTPSCLF QDVDERAAPL LGYLPQDLLG APVLLFLHPE DRPLMLAIHK KILQLAGQPF
C1-1        TTRHTPSCLF QDVDERAAPL LGYLPQDLLG APVLLFLHPE DRPLMLAIHK KILQLAGQPF
C1-2        TTRHTPSCLF QDVDERAAPL LGYLPQDLLG APVLLFLHPE DRPLMLAIHK KILQLAGQPF
            ********** ********** ********** ********** ********** **********
mper1       DHSPIRFCAR NGEYVTMDTS WAGFVHPWSR KVAFVLGRHK VRTAPLNEDV FTPPAPSPAP
C1-1        DHSPIRFCAR KRGICHHGHQ LGRFCAPLEP QGGFRVGSP* ---------- ----------
C1-2        DHSPIRFCAR NMSPWTPAGP VLCTPGAARW LSCWVAIKCA RHP*------ ----------
            **********
mper1       SLDSDIQELS EQIHRLLLQP VHSSSPTGLC GVGPLMSPGP LHSPGSSSDS NGGDAEGPGP
C1-1        ---------- ---------- ---------- ---------- ---------- ----------
C1-2        ---------- ---------- ---------- ---------- ---------- ----------

mper1       PAPVTFQQIC KDVHLVKHQG QQLFIESRAK PPPRPRLLAT GTFKAKVLPC QSPNPELEVA
C1-1        ---------- ---------- ---------- ---------- ---------- ----------
C1-2        ---------- ---------- ---------- ---------- ---------- ----------

mper1       PVPDQASLAL APEEPERKET SGCSYQQINC LDSILRYLES CNIPSTTKRK CASSSSYTAS
C1-1        ---------- ---------- ---------- ---------- ---------- ----------
C1-2        ---------- ---------- ---------- ---------- ---------- ----------

mper1       SASDDDKQRA GPVPVGAKKD PSSAMLSGEG ATPRKEPVVG GTLSPLALAN KAESVVSVTS
C1-1        ---------- ---------- ---------- ---------- ---------- ----------
C1-2        ---------- ---------- ---------- ---------- ---------- ----------

mper1       QCSFSSTIVH VGDKKPPESD IIMMEDLPGL APGPAPSPAP SPTVAPDPTP DAYRPVGLTK
C1-1        ---------- ---------- ---------- ---------- ---------- ----------
C1-2        ---------- ---------- ---------- ---------- ---------- ----------

mper1       AVLSLHTQKE EQAFLNRFRD LGRLRGLDTS SVAPSAPGCH HGPIPPGRRH HCRSKAKRSR
C1-1        ---------- ---------- ---------- ---------- ---------- ----------
C1-2        ---------- ---------- ---------- ---------- ---------- ----------

mper1       HHHHQTPRPE TPCYVSHPSP VPSSGPWPPP PATTPFPAMV QPYPLPVFSP RGGPQPLPPA
C1-1        ---------- ---------- ---------- ---------- ---------- ----------
C1-2        ---------- ---------- ---------- ---------- ---------- ----------

mper1       PTSVSPATFP SPLVTPMVAL VLPNYLFPTP PSYPYGVSQA PVEGPPTPAS HSPSPSLPPP
C1-1        ---------- ---------- ---------- ---------- ---------- ----------
C1-2        ---------- ---------- ---------- ---------- ---------- ----------

mper1       PLSPPHRPDS PLFNSRCSSP LQLNLLQLEE SPRTEGGAAA GGPGSSAGPL PPSEETAEPE
C1-1        ---------- ---------- ---------- ---------- ---------- ----------
C1-2        ---------- ---------- ---------- ---------- ---------- ----------

mper1       ARLVEVTESS NQDALSGSSD LLELLLQEDS RSGTGSAASG SLGSGLGSGS GSGSHEGGST
C1-1        ---------- ---------- ---------- ---------- ---------- ----------
C1-2        ---------- ---------- ---------- ---------- ---------- ----------

mper1       SASITRSSQS SHTSKYFGSI DSSEAEAGAA RARTEPGDQV IKCVLQDPIW LLMANADQRV
C1-1        ---------- ---------- ---------- ---------- ---------- ----------
C1-2        ---------- ---------- ---------- ---------- ---------- ----------

mper1       MMTYQVPSRD AASVLKQDRE RLRAMQKQQP RFSEDQRREL GAVHSWVRKG QLPRALDVTA
C1-1        ---------- ---------- ---------- ---------- ---------- ----------
C1-2        ---------- ---------- ---------- ---------- ---------- ----------

mper1       CVDCGSSVQD PGHSDDPLFS ELDGLGLEPM EEGGGEGGGC GVGGGGGDGG EEAQTQIGAK
C1-1        ---------- ---------- ---------- ---------- ---------- ----------
C1-2        ---------- ---------- ---------- ---------- ---------- ----------

mper1       GSSSQDSAME EEEQGGGSSS PALPAEENST S*
C1-1        ---------- ---------- ---------- --
C1-2        ---------- ---------- ---------- --
```

Figure S12. Predicted translation products of both alleles in the *Per1* deletion cell strain (*Per1*-C1).

```
mper1     MSGPLEGADG GGDPRPGEPF CPGGVPSPGA PQHRPCPGPS LADDTDANSN GSSGNESNGP
B7-1      MSGPLEGADG GGDPRPGEPF CPGGVPSPGA PQHRPCPGPS LADDTDANSN GSSGNESNGP
B7-2      MSGPLEGADG GGDPRPGEPF CPGGVPSPGA PQHRPCPGPS LADDTDANSN GSSGNESNGP
          ********** ********** ********** ********** ********** **********
mper1     ESRGASQRSS HSSSSGNGKD SALLETTESS KSTNSQSPSP PSSSIAYSLL SASSEQDNPS
B7-1      ESRGASQRSS HSSSSGNGKD SALLETTESS KSTNSQSPSP PSSSIAYSLL SASSEQDNPS
B7-2      ESRGASQRSS HSSSSGNGKD SALLETTESS KSTNSQSPSP PSSSIAYSLL SASSEQDNPS
          ********** ********** ********** ********** ********** **********
mper1     TSGCSSEQSA RARTQKELMT ALRELKLRLP PERRGKGRSG TLATLQYALA CVKQVQANQE
B7-1      TSGCSSEQSA RARTQKELMT ALRELKLRLP PERRGKGRSG TLATLQYALA CVKQVQANQE
B7-2      TSGCSSEQSA RARTQKELMT ALRELKLRLP PERRGKGRSG TLATLQYALA CVKQVQANQE
          ********** ********** ********** ********** ********** **********
mper1     YYQQWSLEEG EPCAMDMSTY TLEELEHITS EYTLRNQDTF SVAVSFLTGR IVYISEQAGV
B7-1      YYQQWSLEEG EPCAMDMSTY TLEELEHITS EYTLRNQDTF SVAVSFLTGR IVYISEQAGV
B7-2      YYQQWSLEEG EPCAMDMSTY TLEELEHITS EYTLRNQDTF SVAVSFLTGR IVYISEQAGV
          ********** ********** ********** ********** ********** **********
mper1     LLRCKRDVFR GARFSELLAP QDVGVFYGST TPSRLPTWGT GTSAGSGLKD FTQEKSVFCR
B7-1      LLRCKRDVFR GARFSELLAP QDVGVFYGST TPSRLPTWGT GTSAGSGLKD FTQEKSVFCR
B7-2      LLRCKRDVFR GARFSELLAP QDVGVFYGST TPSRLPTWGT GTSAGSGLKD FTQEKSVFCR
          ********** ********** ********** ********** ********** **********
mper1     IRGGPDRDPG PRYQPFRLTP YVTKIRVSDG APAQPCCLLI AERIHSGYEA PRIPPDKRIF
B7-1      IRGGPDRDPG PRYQPFRLTP YVTKIRVSDG APAQPCCLLI AERIHSGYEA PRIPPDKRIF
B7-2      IRGGPDRDPG PRYQPFRLTP YVTKIRVSDG APAQPCCLLI AERIHSGYEA PRIPPDKRIF
          ********** ********** ********** ********** ********** **********
mper1     TTRHTPSCLF QDVDERAAPL LGYLPQDLLG APVLLFLHPE DRPLMLAIHK KILQLAGQPF
B7-1      TTRHTPSCLF QDVDERAAPL LGYLPQDLLG APVLLFLHPE DRPLMLAIHK KILQLAGQPF
B7-2      TTRHTPSCLF QDVDERAAPL LGYLPQDLLG APVLLFLHPE DRPLMLAIHK KILQLAGQPF
          ********** ********** ********** ********** ********** **********
mper1     DHSPIRFCAR NGEYVTMDTS WAGFVHPWSR KVAFVLGRHK VRTAPLNEDV FTPPAPSPAP
B7-1      DHSPIRFCAR KRGICHHGHQ LGRFCAPLEP QGGFRVGSP* ---------- ----------
B7-2      DHSPIRFCAR NGDMSPWTPA GPVLCTPGAA RWLSCWVAIK CARHP*---- ----------
          **********
mper1     SLDSDIQELS EQIHRLLLQP VHSSSPTGLC GVGPLMSPGP LHSPGSSSDS NGGDAEGPGP
B7-1      ---------- ---------- ---------- ---------- ---------- ----------
B7-2      ---------- ---------- ---------- ---------- ---------- ----------

mper1     PAPVTFQQIC KDVHLVKHQG QQLFIESRAK PPPRPRLLAT GTFKAKVLPC QSPNPELEVA
B7-1      ---------- ---------- ---------- ---------- ---------- ----------
B7-2      ---------- ---------- ---------- ---------- ---------- ----------

mper1     PVPDQASLAL APEEPERKET SGCSYQQINC LDSILRYLES CNIPSTTKRK CASSSSYTAS
B7-1      ---------- ---------- ---------- ---------- ---------- ----------
B7-2      ---------- ---------- ---------- ---------- ---------- ----------

mper1     SASDDDKQRA GPVPVGAKKD PSSAMLSGEG ATPRKEPVVG GTLSPLALAN KAESVVSVTS
B7-1      ---------- ---------- ---------- ---------- ---------- ----------
B7-2      ---------- ---------- ---------- ---------- ---------- ----------

mper1     QCSFSSTIVH VGDKKPPESD IIMMEDLPGL APGPAPSPAP SPTVAPDPTP DAYRPVGLTK
B7-1      ---------- ---------- ---------- ---------- ---------- ----------
B7-2      ---------- ---------- ---------- ---------- ---------- ----------

mper1     AVLSLHTQKE EQAFLNRFRD LGRLRGLDTS SVAPSAPGCH HGPIPPGRRH HCRSKAKRSR
B7-1      ---------- ---------- ---------- ---------- ---------- ----------
B7-2      ---------- ---------- ---------- ---------- ---------- ----------

mper1     HHHHQTPRPE TPCYVSHPSP VPSSGPWPPP PATTPFPAMV QPYPLPVFSP RGGPQPLPPA
B7-1      ---------- ---------- ---------- ---------- ---------- ----------
B7-2      ---------- ---------- ---------- ---------- ---------- ----------

mper1     PTSVSPATFP SPLVTPMVAL VLPNYLFPTP PSYPYGVSQA PVEGPPTPAS HSPSPSLPPP
B7-1      ---------- ---------- ---------- ---------- ---------- ----------
B7-2      ---------- ---------- ---------- ---------- ---------- ----------

mper1     PLSPPHRPDS PLFNSRCSSP LQLNLLQLEE SPRTEGGAAA GGPGSSAGPL PPSEETAEPE
B7-1      ---------- ---------- ---------- ---------- ---------- ----------
B7-2      ---------- ---------- ---------- ---------- ---------- ----------

mper1     ARLVEVTESS NQDALSGSSD LLELLLQEDS RSGTGSAASG SLGSGLGSGS GSGSHEGGST
B7-1      ---------- ---------- ---------- ---------- ---------- ----------
B7-2      ---------- ---------- ---------- ---------- ---------- ----------

mper1     SASITRSSQS SHTSKYFGSI DSSEAEAGAA RARTEPGDQV IKCVLQDPIW LLMANADQRV
B7-1      ---------- ---------- ---------- ---------- ---------- ----------
B7-2      ---------- ---------- ---------- ---------- ---------- ----------

mper1     MMTYQVPSRD AASVLKQDRE RLRAMQKQQP RFSEDQRREL GAVHSWVRKG QLPRALDVTA
B7-1      ---------- ---------- ---------- ---------- ---------- ----------
B7-2      ---------- ---------- ---------- ---------- ---------- ----------

mper1     CVDCGSSVQD PGHSDDPLFS ELDGLGLEPM EEGGGEGGGC GVGGGGGDGG EEAQTQIGAK
B7-1      ---------- ---------- ---------- ---------- ---------- ----------
B7-2      ---------- ---------- ---------- ---------- ---------- ----------

mper1     GSSSQDSAME EEEQGGGSSS PALPAEENST S*
B7-1      ---------- ---------- ---------- --
B7-2      ---------- ---------- ---------- --
```

Figure S13. Predicted translation products of both alleles in the *Per1* deletion cell strain (*Per1*-B7).

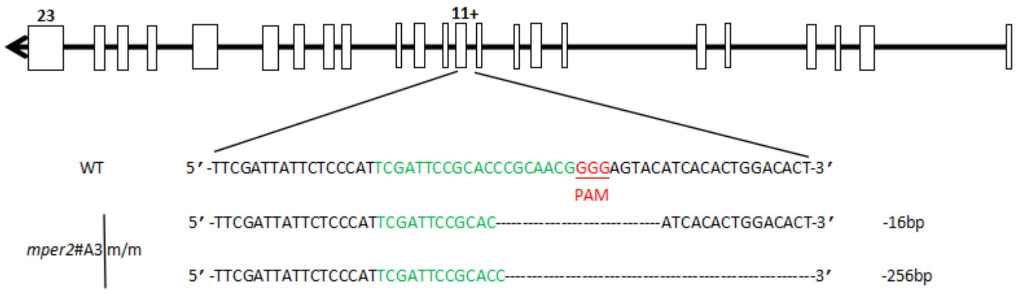

Figure S14. The sgRNA targeting the *Per2* gene and the sequences of both alleles in the *Per2* deletion cell strain.

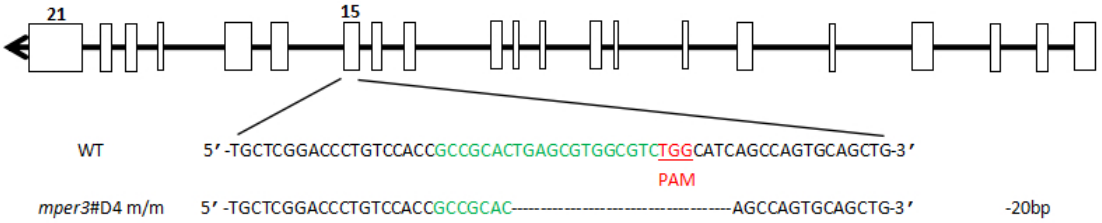

Figure S15. The sgRNA targeting the *Per3* gene and the sequences of both alleles in the *Per3* deletion cell strain.

```
mper3      MDPCGDPAVP GGDCPQTRGP GLQGASGQEG PLQGTCVDSS HSEHEDRNRM SEELIMVVQE
D4-1/2     MDPCGDPAVP GGDCPQTRGP GLQGASGQEG PLQGTCVDSS HSEHEDRNRM SEELIMVVQE
           ********** ********** ********** ********** ********** **********

mper3      MKKYFPAERH TKPSTLDALN YALRCVHSVQ ANSDFFQSLG PRGAHQADVT VYSLEDLTAL
D4-1/2     MKKYFPAERH TKPSTLDALN YALRCVHSVQ ANSDFFQSLG PRGAHQADVT VYSLEDLTAL
           ********** ********** ********** ********** ********** **********

mper3      ASEHTSKNTD TFAAVFSFLS GRLVHISEQA ALILNSKRGF LKSVHFVDLL APQDVRAFYA
D4-1/2     ASEHTSKNTD TFAAVFSFLS GRLVHISEQA ALILNSKRGF LKSVHFVDLL APQDVRAFYA
           ********** ********** ********** ********** ********** **********

mper3      HTAPTQLPFW NNWTQRASQY ECAPAKPFFC RICGGGDREK RHYSPFRILP YLVHVHSSAQ
D4-1/2     HTAPTQLPFW NNWTQRASQY ECAPAKPFFC RICGGGDREK RHYSPFRILP YLVHVHSSAQ
           ********** ********** ********** ********** ********** **********

mper3      PEPEPCCLTL VEKIHSGYEA PRIPVDKRIF TTTHTPGCVF LEVDERAVPL LGYLPQDLIG
D4-1/2     PEPEPCCLTL VEKIHSGYEA PRIPVDKRIF TTTHTPGCVF LEVDERAVPL LGYLPQDLIG
           ********** ********** ********** ********** ********** **********

mper3      TSILTYLHPE DRPLMVAIHQ KVLKYAGHPP FEHSPVRFCT QNGEYVILDS SWSSFVNPWS
D4-1/2     TSILTYLHPE DRPLMVAIHQ KVLKYAGHPP FEHSPVRFCT QNGEYVILDS SWSSFVNPWS
           ********** ********** ********** ********** ********** **********

mper3      RKVSFIIGRH KVRTSPLNED VFATRIKKAA SNDKDIAELQ EQIHKLLLQP VHASASSGYG
D4-1/2     RKVSFIIGRH KVRTSPLNED VFATRIKKAA SNDKDIAELQ EQIHKLLLQP VHASASSGYG
           ********** ********** ********** ********** ********** **********

mper3      SLGSSGSQEQ HVSITSSSES SGHCPEEGQH EQMTLQQVYA SVNKIKNVGQ QLYIESMARS
D4-1/2     SLGSSGSQEQ HVSITSSSES SGHCPEEGQH EQMTLQQVYA SVNKIKNVGQ QLYIESMARS
           ********** ********** ********** ********** ********** **********

mper3      SVKPVAETCV EPQGGDEQKD FSSSQTLKNK STTDTGSGGN LQQEQPSSSY QQMNCIDSVI
D4-1/2     SVKPVAETCV EPQGGDEQKD FSSSQTLKNK STTDTGSGGN LQQEQPSSSY QQMNCIDSVI
           ********** ********** ********** ********** ********** **********

mper3      RYLTSYSLPA LKRKCISCTN TSSSSEEAKP IPEVDSSQRD TEQLLDIRKQ ETTGPSTDIE
D4-1/2     RYLTSYSLPA LKRKCISCTN TSSSSEEAKP IPEVDSSQRD TEQLLDIRKQ GTTGPSTDIE
           ********** ********** ********** ********** ********** **********

mper3      GGAARTLSTA ALSVASGISQ CSCSSTSGHA PPLQSESVAV ACKPWALRTK ASHLAAGGFK
D4-1/2     GGAARTLSTA AQPVQLQQHL WPRSAPTVRK CCRGV*---- ---------- ----------
           ********** *

mper3      HVGLTAAVLS AHTQKEEQNY VDRFREKILT SPYGCYLQQE SRNRAQYSCV QAGSTAKHSR
D4-1/2     ---------- ---------- ---------- ---------- ---------- ----------

mper3      CAGSERQKHK RKKLPAPVDT SSPGAHLCPH VTGLLPDEQH WGPSASPSPL GAGLAFPSAL
D4-1/2     ---------- ---------- ---------- ---------- ---------- ----------

mper3      VVPSQTPYLL PSFPLQDMAS QGVGVSAAWG AAAGCPPLSA GPQAVAAFPS AYVDTLMTIF
D4-1/2     ---------- ---------- ---------- ---------- ---------- ----------

mper3      LHNAPLFPLW PPSFSPYPSL GAAGSSELAP LVPAMAPNPE PTTSGHSQRR VEENWEAHSE
D4-1/2     ---------- ---------- ---------- ---------- ---------- ----------

mper3      ELPFISSRSS SPLQLNLLQE EMPAPSESAD AVRRGAGPDA KHHCVTGPSG SRSRHCTSGE
D4-1/2     ---------- ---------- ---------- ---------- ---------- ----------

mper3      LATATAQQES AAASGSSASS IYFSSTDYAS EVSENRQRPQ DRQRDEALPG AAEESIWRMI
D4-1/2     ---------- ---------- ---------- ---------- ---------- ----------

mper3      ERTPECVLMT YQVPERGREE VLKQDLEKLQ SMEQQQPLFS PAQREELAKV RSWIHSHTAP
D4-1/2     ---------- ---------- ---------- ---------- ---------- ----------

mper3      QEGHLQSCVA CEDRGSVGDT AEVLEQHPAE DTS*
D4-1/2     ---------- ---------- ---------- ----
```

Figure S16. Predicted translation products of both alleles in the *Per3* deletion cell strain.

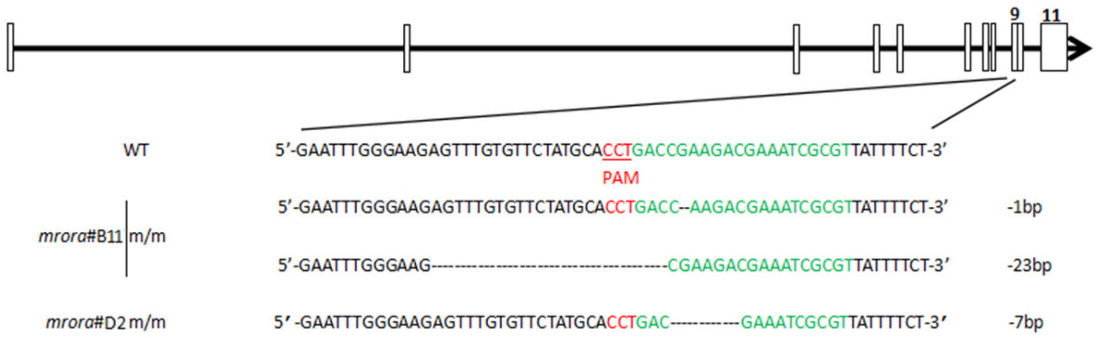

Figure S17. The sgRNA targeting the *Rora* gene and the sequences of both alleles in the *Rora* deletion cell strain.

```
mrora       MESAPAAPDP AASEPGSSGS EAAAGSRETP LTQDTGRKSE APGAGRRQSY ASSSRGISVT
B11-1       MESAPAAPDP AASEPGSSGS EAAAGSRETP LTQDTGRKSE APGAGRRQSY ASSSRGISVT
B11-2       MESAPAAPDP AASEPGSSGS EAAAGSRETP LTQDTGRKSE APGAGRRQSY ASSSRGISVT
            ********** ********** ********** ********** ********** **********

mrora       KKTHTSQIEI IPCKICGDKS SGIHYGVITC EGCKGFFRRS QQSNATYSCP RQKNCLIDRT
B11-1       KKTHTSQIEI IPCKICGDKS SGIHYGVITC EGCKGFFRRS QQSNATYSCP RQKNCLIDRT
B11-2       KKTHTSQIEI IPCKICGDKS SGIHYGVITC EGCKGFFRRS QQSNATYSCP RQKNCLIDRT
            ********** ********** ********** ********** ********** **********

mrora       SRNRCQHCRL QKCLAVGMSR DAVKFGRMSK KQRDSLYAEV QKHRMQQQQR DHQQQPGEAE
B11-1       SRNRCQHCRL QKCLAVGMSR DAVKFGRMSK KQRDSLYAEV QKHRMQQQQR DHQQQPGEAE
B11-2       SRNRCQHCRL QKCLAVGMSR DAVKFGRMSK KQRDSLYAEV QKHRMQQQQR DHQQQPGEAE
            ********** ********** ********** ********** ********** **********

mrora       PLTPTYNISA NGLTELHDDL STYMDGHTPE GSKADSAVSS FYLDIQPSPD QSGLDINGIK
B11-1       PLTPTYNISA NGLTELHDDL STYMDGHTPE GSKADSAVSS FYLDIQPSPD QSGLDINGIK
B11-2       PLTPTYNISA NGLTELHDDL STYMDGHTPE GSKADSAVSS FYLDIQPSPD QSGLDINGIK
            ********** ********** ********** ********** ********** **********

mrora       PEPICDYTPA SGFFPYCSFT NGETSPTVSM AELEHLAQNI SKSHLETCQY LREELQQITW
B11-1       PEPICDYTPA SGFFPYCSFT NGETSPTVSM AELEHLAQNI SKSHLETCQY LREELQQITW
B11-2       PEPICDYTPA SGFFPYCSFT NGETSPTVSM AELEHLAQNI SKSHLETCQY LREELQQITW
            ********** ********** ********** ********** ********** **********

mrora       QTFLQEEIEN YQNKQREVMW QLCAIKITEA IQYVVEFAKR IDGFMELCQN DQIVLLKAGS
B11-1       QTFLQEEIEN YQNKQREVMW QLCAIKITEA IQYVVEFAKR IDGFMELCQN DQIVLLKAGS
B11-2       QTFLQEEIEN YQNKQREVMW QLCAIKITEA IQYVVEFAKR IDGFMELCQN DQIVLLKAGS
            ********** ********** ********** ********** ********** **********

mrora       LEVVFIRMCR AFDSQNNTVY FDGKYASPDV FKSLGCEDFI SFVFEFGKSL CSMHLTEDEI
B11-1       LEVVFIRMCR AFDSQNNTVY FDGKYASPDV FKSLGCEDFI SFVFEFGKRR RNRVIFCIRT
B11-2       LEVVFIRMCR AFDSQNNTVY FDGKYASPDV FKSLGCEDFI SFVFEFGKSL CSMHLTKTKS
            ********** ********** ********** ********** ********

mrora       ALFSAFVLMS ADRSWLQEKV KIEKLQQKIQ LALQHVLQKN HREDGILTKL ICKVSTLRAL
B11-1       DVSGSLVASG KGKNRKAATE NSAGPSARPT EEPPRRWNSN QANMQGVYVK SPMWTTYGKA
B11-2       RYFLHSY*-- ---------- ---------- ---------- ---------- ----------

mrora       CGRHTEKLMA FKAIYPDIVR LHFPPLYKEL FTSEFEPAMQ IDG*
B11-1       NGI*------ ---------- ---------- ---------- ----
B11-2       ---------- ---------- ---------- ---------- ----.
```

Figure S18. Predicted translation products of both alleles in the *Rora* deletion cell strain (*Rora*-B11).

```
mrora      MESAPAAPDP AASEPGSSGS EAAAGSRETP LTQDTGRKSE APGAGRRQSY ASSSRGISVT
D2-1/2     MESAPAAPDP AASEPGSSGS EAAAGSRETP LTQDTGRKSE APGAGRRQSY ASSSRGISVT
           ********** ********** ********** ********** ********** **********

mrora      KKTHTSQIEI IPCKICGDKS SGIHYGVITC EGCKGFFRRS QQSNATYSCP RQKNCLIDRT
D2-1/2     KKTHTSQIEI IPCKICGDKS SGIHYGVITC EGCKGFFRRS QQSNATYSCP RQKNCLIDRT
           ********** ********** ********** ********** ********** **********

mrora      SRNRCQHCRL QKCLAVGMSR DAVKFGRMSK KQRDSLYAEV QKHRMQQQQR DHQQQPGEAE
D2-1/2     SRNRCQHCRL QKCLAVGMSR DAVKFGRMSK KQRDSLYAEV QKHRMQQQQR DHQQQPGEAE
           ********** ********** ********** ********** ********** **********

mrora      PLTPTYNISA NGLTELHDDL STYMDGHTPE GSKADSAVSS FYLDIQPSPD QSGLDINGIK
D2-1/2     PLTPTYNISA NGLTELHDDL STYMDGHTPE GSKADSAVSS FYLDIQPSPD QSGLDINGIK
           ********** ********** ********** ********** ********** **********

mrora      PEPICDYTPA SGFFPYCSFT NGETSPTVSM AELEHLAQNI SKSHLETCQY LREELQQITW
D2-1/2     PEPICDYTPA SGFFPYCSFT NGETSPTVSM AELEHLAQNI SKSHLETCQY LREELQQITW
           ********** ********** ********** ********** ********** **********

mrora      QTFLQEEIEN YQNKQREVMW QLCAIKITEA IQYVVEFAKR IDGFMELCQN DQIVLLKAGS
D2-1/2     QTFLQEEIEN YQNKQREVMW QLCAIKITEA IQYVVEFAKR IDGFMELCQN DQIVLLKAGS
           ********** ********** ********** ********** ********** **********

mrora      LEVVFIRMCR AFDSQNNTVY FDGKYASPDV FKSLGCEDFI SFVFEFGKSL CSMHLTEDEI
D2-1/2     LEVVFIRMCR AFDSQNNTVY FDGKYASPDV FKSLGCEDFI SFVFEFGKSL CSMHLTKSRY
           ********** ********** ********** ********** ********** ******

mrora      ALFSAFVLMS ADRSWLQEKV KIEKLQQKIQ LALQHVLQKN HREDGILTKL ICKVSTLRAL
D2-1/2     FLHSY*---- ---------- ---------- ---------- ---------- ----------

mrora      CGRHTEKLMA FKAIYPDIVR LHFPPLYKEL FTSEFEPAMQ IDG*
D2-1/2     ---------- ---------- ---------- ---------- ----
```

Figure S19. Predicted translation products of both alleles in the *Rora* deletion cell strain (*Rora*-D2).

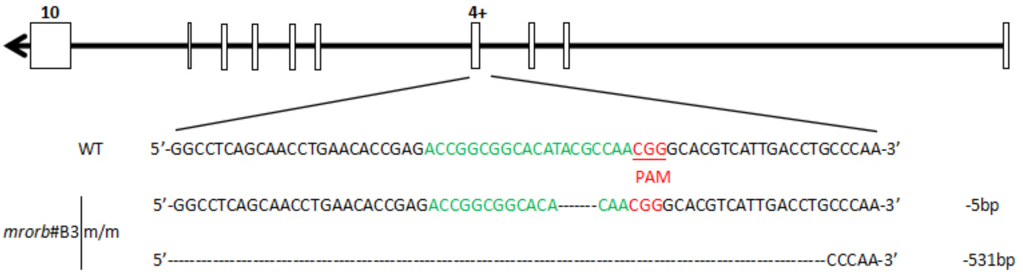

Figure S20. The sgRNA targeting the *Rorb* gene and the sequences of both alleles in the *Rorb* deletion cell strain.

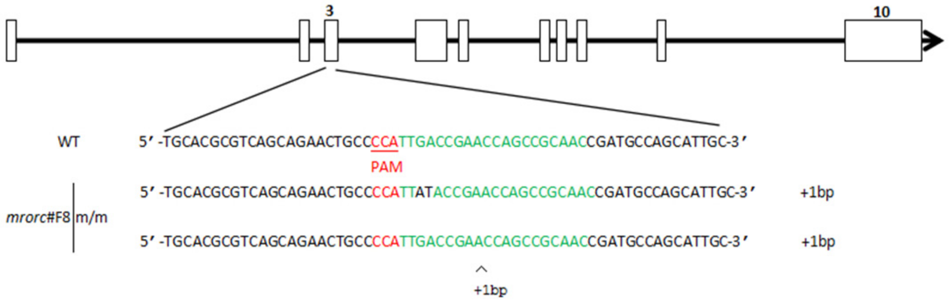

Figure S21. The sgRNA targeting the *Rorc* gene and the sequences of both alleles in the *Rorc* deletion cell strain.

```
mrorc      MRTQIEVIPC KICGDKSSGI HYGVITCEGC KGFFRRSQQC NVAYSCTRQQ NCPIDRTSRN
F8-1       MRTQIEVIPC KICGDKSSGI HYGVITCEGC KGFFRRSQQC NVAYSCTRQQ NCPIIPNQPQ
F8-2       MRTQIEVIPC KICGDKSSGI HYGVITCEGC KGFFRRSQQC NVAYSCTRQQ NCPIEPNQPQ
           *********v ********** ********** ********** ********** ****------

mrorc      RCQHCRLQKC LALGMSRDAV KFGRMSKKQR DSLHAEVQKQ LQQQQQQEQV AKTPPAGSRG
F8-1       PMPALPPAEV PGSGHVPRCC QVWPNVQEAE GQSTCRSAET TATAAATGTS GQDSSSWEPR
F8-2       PMPALPPAEV PGSGHVPRCC QVWPNVQEAE GQSTCRSAET TATAAATGTS GQDSSSWEPR

mrorc      ADTLTYTLGL SDGQLPLGAS PDLPEASACP PGLLRASGSG PPYSNTLAKT EVQGASCHLE
F8-1       SRHTYIHFRA LRWAATTGRL T*-------- ---------- ---------- ----------
F8-2       SRHTYIHFRA LRWAATTGRL T*-------- ---------- ---------- ----------

mrorc      YSPERGKAEG RDSIYSTDGQ LTLGRCGLRF EETRHPELGE PEQGPDSHCI PSFCSAPEVP
F8-1       ---------- ---------- ---------- ---------- ---------- ----------
F8-2       ---------- ---------- ---------- ---------- ---------- ----------

mrorc      YASLTDIEYL VQNVCKSFRE TCQLRLEDLL RQRTNLFSRE EVTSYQRKSM WEMWERCAHH
F8-1       ---------- ---------- ---------- ---------- ---------- ----------
F8-8       ---------- ---------- ---------- ---------- ---------- ----------

mrorc      LTEAIQYVVE FAKRLSGFME LCQNDQIILL KAGAMEVVLV RMCRAYNANN HTVFFEGKYG
F8-1       ---------- ---------- ---------- ---------- ---------- ----------
F8-8       ---------- ---------- ---------- ---------- ---------- ----------

mrorc      LTEAIQYVVE FAKRLSGFME LCQNDQIILL KAGAMEVVLV RMCRAYNANN HTVFFEGKYG
F8-1       ---------- ---------- ---------- ---------- ---------- ----------
F8-2       ---------- ---------- ---------- ---------- ---------- ----------

mrorc      GVELFRALGC SELISSIFDF SHFLSALCFS EDEIALYTAL VLINANRPGL QEKRRVEHLQ
F8-1       ---------- ---------- ---------- ---------- ---------- ----------
F8-2       ---------- ---------- ---------- ---------- ---------- ----------

mrorc      GVELFRALGC SELISSIFDF SHFLSALCFS EDEIALYTAL VLINANRPGL QEKRRVEHLQ
F8-1       ---------- ---------- ---------- ---------- ---------- ----------
F8-2       ---------- ---------- ---------- ---------- ---------- ----------

mrorc      YNLELAFHHH LCKTHRQGLL AKLPPKGKLR SLCSQHVEKL QIFQHLHPIV VQAAFPPLYK
F8-1       ---------- ---------- ---------- ---------- ---------- ----------
F8-2       ---------- ---------- ---------- ---------- ---------- ----------

mrorc      ELFSTDVESP EGLSK*
F8-1       ---------- ------
F8-2       ---------- ------
```

Figure S22. Predicted translation products of both alleles in the *Rorc* deletion cell strain.

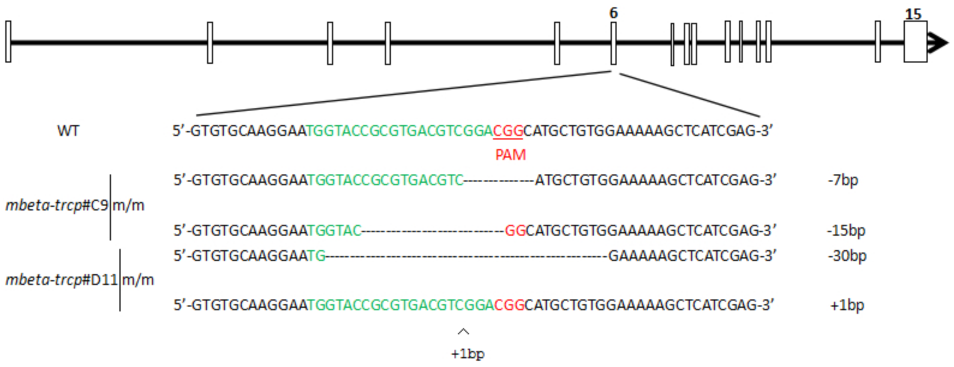

Figure S23. The sgRNA targeting the *Beta-trcp* gene and the sequences of both alleles in the *Beta-trcp* deletion cell strain.

```
mbeta-trcp   MDPAEAVLQE KALKFMCSMP RSLWLGCSSL ADSMPSLRCL YNPGTGALTA FQNSSEREDC
C9-1         MDPAEAVLQE KALKFMCSMP RSLWLGCSSL ADSMPSLRCL YNPGTGALTA FQNSSEREDC
C9-2         MDPAEAVLQE KALKFMCSMP RSLWLGCSSL ADSMPSLRCL YNPGTGALTA FQNSSEREDC
             ********** ********** ********** ********** ********** **********

mbeta-trcp   NNGEPPRKII PEKNSLRQTY NSCARLCINQ ETVCLTSTAM KTENCVAKAK LANGTSSMIV
C9-1         NNGEPPRKII PEKNSLRQTY NSCARLCINQ ETVCLTSTAM KTENCVAKAK LANGTSSMIV
C9-2         NNGEPPRKII PEKNSLRQTY NSCARLCINQ ETVCLTSTAM KTENCVAKAK LANGTSSMIV
             ********** *********  ********** ********** ********** **********

mbeta-trcp   PKQRKLSASY EKEKELCVKY FEQWSESDQV EFVEHLISQM CHYQHGHINS YLKPMLQRDF
C9-1         PKQRKLSASY EKEKELCVKY FEQWSESDQV EFVEHLISQM CHYQHGHINS YLKPMLQRDF
C9-2         PKQRKLSASY EKEKELCVKY FEQWSESDQV EFVEHLISQM CHYQHGHINS YLKPMLQRDF
             ********** *********  ********** ********** ********** **********

mbeta-trcp   ITALPARGLD HIAENILSYL DAKSLCAAEL VCKEWYRVTS DGMLWKKLIE RMVRTDSLWR
C9-1         ITALPARGLD HIAENILSYL DAKSLCAAEL VCKEWYRVTS CCGKSSSRGW SGRTLCGEAW
C9-2         ITALPARGLD HIAENILSYL DAKSLCAAEL VCKEWY---- -GMLWKKLIE RMVRTDSLWR
             ********** *********  ********** ******

mbeta-trcp   GLAERRGWGQ YLFKNKPPDE NAPPNSFYRA LYPKIIQDIE TIESNWRCGR HSLQRIHCRS
C9-1         QSAEAGDSTY SKTNLLMRTL LPTPFIERFI LKSYKTLRQ* ---------- ----------
C9-2         GLAERRGWGQ YLFKNKPPDE NAPPNSFYRA LYPKIIQDIE TIESNWRCGR HSLQRIHCRS

mbeta-trcp   ETSKGVYCLQ YDDQKIVSGL RDNTIKIWDK STLECKRILT GHTGSVLCLQ YDERVIITGS
C9-1         ---------- ---------- ---------- ---------- ---------- ----------
C9-2         ETSKGVYCLQ YDDQKIVSGL RDNTIKIWDK STLECKRILT GHTGSVLCLQ YDERVIITGS

mbeta-trcp   SDSTVRVWDV NAGEMLNTLI HHCEAVLHLR FNNGMMVTCS KDRSIAVWDM ASPTDITLRR
C9-1         ---------- ---------- ---------- ---------- ---------- ----------
C9-2         SDSTVRVWDV NAGEMLNTLI HHCEAVLHLR FNNGMMVTCS KDRSIAVWDM ASPTDITLRR

mbeta-trcp   VLVGHRAAVN VVDFDDKYIV SASGDRTIKV WNTSTCEFVR TLNGHKRGIA CLQYRDRLVV
C9-1         ---------- ---------- ---------- ---------- ---------- ----------
C9-2         VLVGHRAAVN VVDFDDKYIV SASGDRTIKV WNTSTCEFVR TLNGHKRGIA CLQYRDRLVV

mbeta-trcp   SGSSDNTIRL WDIECGACLR VLEGHEELVR CIRFDNKRIV SGAYDGKIKV WDLMAALDPR
C9-1         ---------- ---------- ---------- ---------- ---------- ----------
C9-2         SGSSDNTIRL WDIECGACLR VLEGHEELVR CIRFDNKRIV SGAYDGKIKV WDLMAALDPR

mbeta-trcp   APAGTLCLRT LVEHSGRVFR LQFDEFQIVS SSHDDTILIW DFLNDPAAHA EPPRSPSRTY
C9-1         ---------- ---------- ---------- ---------- ---------- ----------
C9-2         APAGTLCLRT LVEHSGRVFR LQFDEFQIVS SSHDDTILIW DFLNDPAAHA EPPRSPSRTY

mbeta-trcp   TYISR*
C9-1         ------
C9-2         TYISR*
```

Figure S24. Predicted translation products of both alleles in the *Beta-trcp* deletion cell (C9).

```
mbeta-trcp   MDPAEAVLQE KALKFMCSMP RSLWLGCSSL ADSMPSLRCL YNPGTGALTA FQNSSEREDC
D11-1        MDPAEAVLQE KALKFMCSMP RSLWLGCSSL ADSMPSLRCL YNPGTGALTA FQNSSEREDC
D11-2        MDPAEAVLQE KALKFMCSMP RSLWLGCSSL ADSMPSLRCL YNPGTGALTA FQNSSEREDC
             ********** ********** ********** ********** ********** **********

mbeta-trcp   NNGEPPRKII PEKNSLRQTY NSCARLCINQ ETVCLTSTAM KTENCVAKAK LANGTSSMIV
D11-1        NNGEPPRKII PEKNSLRQTY NSCARLCINQ ETVCLTSTAM KTENCVAKAK LANGTSSMIV
D11-2        NNGEPPRKII PEKNSLRQTY NSCARLCINQ ETVCLTSTAM KTENCVAKAK LANGTSSMIV
             ********** ********** ********** ********** ********** **********

mbeta-trcp   PKQRKLSASY EKEKELCVKY FEQWSESDQV EFVEHLISQM CHYQHGHINS YLKPMLQRDF
D11-1        PKQRKLSASY EKEKELCVKY FEQWSESDQV EFVEHLISQM CHYQHGHINS YLKPMLQRDF
D11-2        PKQRKLSASY EKEKELCVKY FEQWSESDQV EFVEHLISQM CHYQHGHINS YLKPMLQRDF
             ********** ********** ********** ********** ********** **********

mbeta-trcp   ITALPARGLD HIAENILSYL DAKSLCAAEL VCKEWYRVTS DGMLWKKLIE RMVRTDSLWR
D11-1        ITALPARGLD HIAENILSYL DAKSLCAAEL VCKEW----- -----KKLIE RMVRTDSLWR
D11-2        ITALPARGLD HIAENILSYL DAKSLCAAEL VCKEWYRVTS GRHAVEKAHR EDGQDGLSVA
             ********** ********** ********** *****

mbeta-trcp   GLAERRGWGQ YLFKNKPPDE NAPPNSFYRA LYPKIIQDIE TIESNWRCGR HSLQRIHCRS
D11-1        GLAERRGWGQ YLFKNKPPDE NAPPNSFYRA LYPKIIQDIE TIESNWRCGR HSLQRIHCRS
D11-2        RPGRAQRLGT VLIQKQTS*- ---------- ---------- ---------- ----------

mbeta-trcp   ETSKGVYCLQ YDDQKIVSGL RDNTIKIWDK STLECKRILT GHTGSVLCLQ YDERVIITGS
D11-1        ETSKGVYCLQ YDDQKIVSGL RDNTIKIWDK STLECKRILT GHTGSVLCLQ YDERVIITGS
D11-2        ---------- ---------- ---------- ---------- ---------- ----------

mbeta-trcp   SDSTVRVWDV NAGEMLNTLI HHCEAVLHLR FNNGMMVTCS KDRSIAVWDM ASPTDITLRR
D11-1        SDSTVRVWDV NAGEMLNTLI HHCEAVLHLR FNNGMMVTCS KDRSIAVWDM ASPTDITLRR
D11-2        ---------- ---------- ---------- ---------- ---------- ----------

mbeta-trcp   VLVGHRAAVN VVDFDDKYIV SASGDRTIKV WNTSTCEFVR TLNGHKRGIA CLQYRDRLVV
D11-1        VLVGHRAAVN VVDFDDKYIV SASGDRTIKV WNTSTCEFVR TLNGHKRGIA CLQYRDRLVV
D11-2        ---------- ---------- ---------- ---------- ---------- ----------

mbeta-trcp   SGSSDNTIRL WDIECGACLR VLEGHEELVR CIRFDNKRIV SGAYDGKIKV WDLMAALDPR
D11-1        SGSSDNTIRL WDIECGACLR VLEGHEELVR CIRFDNKRIV SGAYDGKIKV WDLMAALDPR
D11-2        ---------- ---------- ---------- ---------- ---------- ----------

mbeta-trcp   APAGTLCLRT LVEHSGRVFR LQFDEFQIVS SSHDDTILIW DFLNDPAAHA EPPRSPSRTY
D11-1        APAGTLCLRT LVEHSGRVFR LQFDEFQIVS SSHDDTILIW DFLNDPAAHA EPPRSPSRTY
D11-2        ---------- ---------- ---------- ---------- ---------- ----------

mbeta-trcp   TYISR*
D11-1        TYISR*
D11-2        ------
```

Figure S25. Predicted translation products of both alleles in the *Beta-trcp* deletion cell (D11).

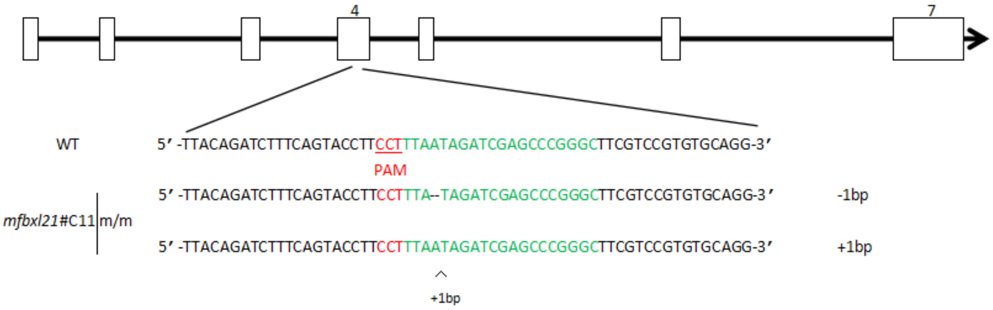

Figure S26. The sgRNA targeting the *Fbxl21* gene and the sequences of both alleles in the *Fbxl21* deletion cell strain.

```
mfbxl21    MKRNNFSAVN KVVQSSPVVK QPKRGLCSSL RQTHALSVLL DWGTLPHHVI LQIFQYLPLI
C11-1      MKRNNFSAVN KVVQSSPVVK QPKRGLCSSL RQTHALSVLL DWGTLPHHVI LQIFQYLPLN
C11-2      MKRNNFSAVN KVVQSSPVVK QPKRGLCSSL RQTHALSVLL DWGTLPHHVI LQIFQYLPL*
           ********** ********** ********** ********** ********** *********

mfbxl21    DRARASSVCR RWNEVFHIPD LWRKFEFELN QSATSYFKST HPDLIQQIIK KHAAHLQYVS
C11-1      RSSPGFVRVQ EME*------ ---------- ---------- ---------- ----------
C11-2      ---------- ---------- ---------- ---------- ---------- ----------

mfbxl21    FKVDSSTESA EAACDILSQL VNCSIQTLGL ISTAKPSFMN VPKSHFVSAL TVVFVNSKSL
C11-1      ---------- ---------- ---------- ---------- ---------- ----------
C11-2      ---------- ---------- ---------- ---------- ---------- ----------

mfbxl21    SSIKIEDTPV DDPSLKILVA NNSDTLRLLK MSSCPHVSSD GILCVADHCQ GLRELALNYY
C11-1      ---------- ---------- ---------- ---------- ---------- ----------
C11-2      ---------- ---------- ---------- ---------- ---------- ----------

mfbxl21    ILSDEILLAL SSETHVNLEH LRIDVVSENP GQIKFHSIKK RSWDALIKHS PRVNVVMYFF
C11-1      ---------- ---------- ---------- ---------- ---------- ----------
C11-2      ---------- ---------- ---------- ---------- ---------- ----------

mfbxl21    LYEEEFEAFF KEETPVTHLY FGRSVSRAIL GRIGLNCPRL IELVVCANGL LPLDSELIRI
C11-1      ---------- ---------- ---------- ---------- ---------- ----------
C11-2      ---------- ---------- ---------- ---------- ---------- ----------


C11-1      ---------- ---------- ---------- ---------- ---------- ----------
C11-2      ---------- ---------- ---------- ---------- ---------- ----------

mfbxl21    LYEEEFEAFF KEETPVTHLY FGRSVSRAIL GRIGLNCPRL IELVVCANGL LPLDSELIRI
C11-1      ---------- ---------- ---------- ---------- ---------- ----------
C11-2      ---------- ---------- ---------- ---------- ---------- ----------

mfbxl21    AKHCKNLTSL GLSECEVSCS AFVEFVRLCG RRLTQLSIME EVLVPDDRYT PDEVHTEVSK
C11-1      ---------- ---------- ---------- ---------- ---------- ----------
C11-2      ---------- ---------- ---------- ---------- ---------- ----------

mfbxl21    HLGRVWFPDV MPIW*
C11-1      ---------- -----
C11-2      ---------- -----
```

Figure S27. Predicted translation products of both alleles in the *Fbxl21* deletion cell.

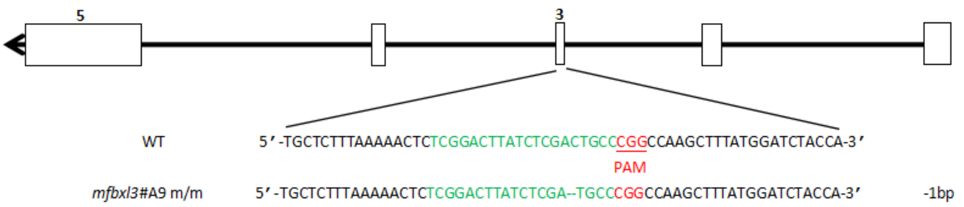

Figure S28. The sgRNA targeting the *Fbxl3* gene and the sequences of both alleles in the *Fbxl3* deletion cell strain.

```
mfbxl3    MKRGGRDSDQ DSAEEGTAEK PKRPRTTQER SQPCDWGNLL QDIVLHVFKY LPLLDRAHAS
F7-1/2    MKRGGRDSDQ DSAEEGTAEK PKRPRTTQER SQPCDWGNLL QDIVLHVFKY LPLLDRAHAS
          ********** ********** ********** ********** ********** **********
mfbxl3    QVCRNWNQVF HMPDLWRCFE FELNQPATSY LKATHPELIK QIIKRHSNHL QYVSFKVDSS
F7-1/2    QVCRNWNQVF HMPDLWRCFE FELNQPATSY LKATHPELIK QIIKRHSNHL QYVSFKVDSS
          ********** ********** ********** ********** ********** **********

mfbxl3    KESAEAACDI LSQLVNCSLK TLGLISTARP SFMDLPKSHF ISALTVVFVN SKSLSSLKID
F7-1/2    KESAEAACDI LSQLVNCSLK TLGLISMPGQ ALWIYQSLTL SLH*------ ----------
          ********** ********** ******

mfbxl3    DTPVDDPSLK VLVANNSDTL KLLKMSSCPH VSPAGILCVA DQCHGLRELA LNYHLLSDEL
F7-1/2    ---------- ---------- ---------- ---------- ---------- ----------

mfbxl3    LLALSSEKHV RLEHLRIDVV SENPGQTHFH TIQKSSWDAF IKHSPKVNLV MYFFLYEEEF
F7-1/2    ---------- ---------- ---------- ---------- ---------- ----------

mfbxl3    DPFFRYEIPA THLYFGRSVS KDVLGRVGMT CPRLVELVVC ANGLRPLDEE LIRIAERCKN
F7-1/2    ---------- ---------- ---------- ---------- ---------- ----------

mfbxl3    LSAIGLGECE VSCSAFVEFV KMCGGRLSQL SIMEEVLIPD QKYSLEQIHW EVSKHLGRVW
F7-1/2    ---------- ---------- ---------- ---------- ---------- ----------

mfbxl3    FPDMMPTW*
F7-1/2    ---------
```

Figure S29. Predicted translation products of both alleles in the *Fbxl3* deletion cell.

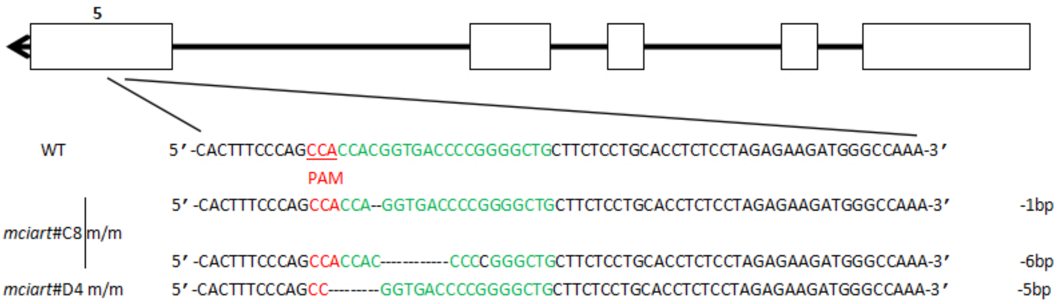

Figure S30. The sgRNA targeting the *Ciart* gene and the sequences of both alleles in the *Ciart* deletion cell strain.

```
mciart    MDSPSSVSSY SSSSLSPSFS TSSVNSDFSF PSDNEREGKG THELRPDTVG QRGGSRPSPG
C8-1      MDSPSSVSSY SSSSLSPSFS TSSVNSDFSF PSDNEREGKG THELRPDTVG QRGGSRPSPG
C8-2      MDSPSSVSSY SSSSLSPSFS TSSVNSDFSF PSDNEREGKG THELRPDTVG QRGGSRPSPG
          ********** ********** ********** ********** ********** **********

mciart    PIRCRHRPRV SSNQHTAPHL EQQGSEVKRS RDGEQETSLN TQGCTTEGDL LFAQKCKELQ
C8-1      PIRCRHRPRV SSNQHTAPHL EQQGSEVKRS RDGEQETSLN TQGCTTEGDL LFAQKCKELQ
C8-2      PIRCRHRPRV SSNQHTAPHL EQQGSEVKRS RDGEQETSLN TQGCTTEGDL LFAQKCKELQ
          ********** ********** ********** ********** ********** **********

mciart    GFIRPLTDLL NGLKMGRFDR GLSSFQQSVA MDRIQRIVGV LQKPQMGERY LGTLLQVEGM
C8-1      GFIRPLTDLL NGLKMGRFDR GLSSFQQSVA MDRIQRIVGV LQKPQMGERY LGTLLQVEGM
C8-2      GFIRPLTDLL NGLKMGRFDR GLSSFQQSVA MDRIQRIVGV LQKPQMGERY LGTLLQVEGM
          ********** ********** ********** ********** ********** **********

mciart    LKTWFPHIAA QKSSSGGSRH QISKHFPSHH GDPGAASPAP LLEKMGQTQL GHLVLKPKQP
C8-1      LKTWFPHIAA QKSSSGGSRH QISKHFPSHH --PRAASPAP LLEKMGQTQL GHLVLKPKQP
C8-2      LKTWFPHIAA QKSSSGGSRH QISKHFPSHQ VTPGLLLLHL S*-------- ----------
          ********** ********** **********

mciart    WHLTGWPAMN LTWIHSTPIC NPPLSSQGSA SGHSPIGTGA SIGVILVLQK GGQPFTHSAP
C8-1      WHLTGWPAMN LTWIHSTPIC NPPLSSQGSA SGHSPIGTGA SIGVILVLQK GGQPFTHSAP
C8-2      ---------- ---------- ---------- ---------- ---------- ----------

mciart    GTPVPPTPLS PVVPGDLKKL PGEEPRCHSL PVTLPSDWSC ILCPPVLPTT DREMTKGHPE
C8-1      GTPVPPTPLS PVVPGDLKKL PGEEPRCHSL PVTLPSDWSC ILCPPVLPTT DREMTKGHPE
C8-2      ---------- ---------- ---------- ---------- ---------- ----------

mciart    PQMTSHPPVA PDPQP*
C8-1      PQMTSHPPVA PDPQP*
C8-2      ---------- ------
```

Figure S31. Predicted translation products of both alleles in the *Ciart* deletion cell (C8).

```
mciart     MDSPSSVSSY SSSSLSPSFS TSSVNSDFSF PSDNEREGKG THELRPDTVG QRGGSRPSPG
D4-1/2     MDSPSSVSSY SSSSLSPSFS TSSVNSDFSF PSDNEREGKG THELRPDTVG QRGGSRPSPG
           ********** ********** ********** ********** ********** **********
mciart     PIRCRHRPRV SSNQHTAPHL EQQGSEVKRS RDGEQETSLN TQGCTTEGDL LFAQKCKELQ
D4-1/2     PIRCRHRPRV SSNQHTAPHL EQQGSEVKRS RDGEQETSLN TQGCTTEGDL LFAQKCKELQ
           ********** ********** ********** ********** ********** **********
mciart     GFIRPLTDLL NGLKMGRFDR GLSSFQQSVA MDRIQRIVGV LQKPQMGERY LGTLLQVEGM
D4-1/2     GFIRPLTDLL NGLKMGRFDR GLSSFQQSVA MDRIQRIVGV LQKPQMGERY LGTLLQVEGM
           ********** ********** ********** ********** ********** **********
mciart     LKTWFPHIAA QKSSSGGSRH QISKHFPSHH GDPGAASPAP LLEKMGQTQL GHLVLKPKQP
D4-1/2     LKTWFPHIAA QKSSSGGSRH QISKHFPSR* ---------- ---------- ----------
           ********** ********** ********
mciart     WHLTGWPAMN LTWIHSTPIC NPPLSSQGSA SGHSPIGTGA SIGVILVLQK GGQPFTHSAP
D4-1/2     ---------- ---------- ---------- ---------- ---------- ----------

mciart     GTPVPPTPLS PVVPGDLKKL PGEEPRCHSL PVTLPSDWSC ILCPPVLPTT DREMTKGHPE
D4-1/2     ---------- ---------- ---------- ---------- ---------- ----------

mciart     PQMTSHPPVA PDPQP*
D4-1/2     ---------- -----
```

Figure S32. Predicted translation products of both alleles in the *Ciart* deletion cell (D4).

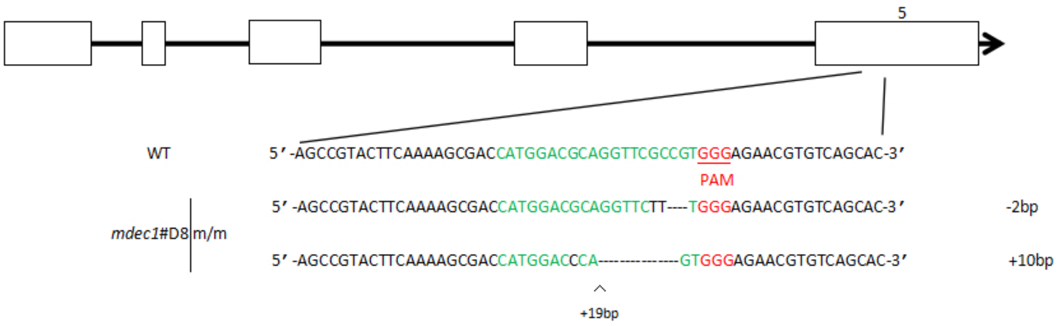

Figure S33. The sgRNA targeting the *Dec1* gene and the sequences of both alleles in the *Dec1* deletion cell strain.

```
mdec1    MERIPSAQPP PTCLPKAPGL EHGDLSGMDF AHMYQVVYKSR RGIKRSEDSK ETYKLPHRLI
D8-1     MERIPSAQPP PTCLPKAPGL EHGDLSGMDF AHMYQVVYKSR RGIKRSEDSK ETYKLPHRLI
D8-2     MERIPSAQPP PTCLPKAPGL EHGDLSGMDF AHMYQVVYKSR RGIKRSEDSK ETYKLPHRLI
         ********** ********** ********** ********** ********** **********

mdec1    EKKRRDRINE CIAQLKDLLP EHLKLTTLGH LEKAVVLELT LKHVKALTNL IDQQQQKIIA
D8-1     EKKRRDRINE CIAQLKDLLP EHLKLTTLGH LEKAVVLELT LKHVKALTNL IDQQQQKIIA
D8-2     EKKRRDRINE CIAQLKDLLP EHLKLTTLGH LEKAVVLELT LKHVKALTNL IDQQQQKIIA
         ********** ********** ********** ********** ********** **********

mdec1    LQSGLQAGDL SGRNLEAGQE MFCSGFQTCA REVLQYLAKH ENTRDLKSSQ LVTHLHRVVS
D8-1     LQSGLQAGDL SGRNLEAGQE MFCSGFQTCA REVLQYLAKH ENTRDLKSSQ LVTHLHRVVS
D8-2     LQSGLQAGDL SGRNLEAGQE MFCSGFQTCA REVLQYLAKH ENTRDLKSSQ LVTHLHRVVS
         ********** ********** ********** ********** ********** **********

mdec1    ELLQGGASRK PLDSAPKAVD LKEKPSFLAK GSEGPGKNCV PVIQRTFAPS GGEQSGSDTD
D8-1     ELLQGGASRK PLDSAPKAVD LKEKPSFLAK GSEGPGKNCV PVIQRTFAPS GGEQSGSDTD
D8-2     ELLQGGASRK PLDSAPKAVD LKEKPSFLAK GSEGPGKNCV PVIQRTFAPS GGEQSGSDTD
         ********** ********** ********** ********** ********** **********

mdec1    TDSGYGGELE KGDLRSEQPY FKSDHGRRFA VGERVSTIKQ ESEEPPTKKS RMQLSEEEGH
D8-1     TDSGYGGELE KGDLRSEQPY FKSDHGRRFF GRTCQHN*-- ---------- ----------
D8-2     TDSGYGGELE KGDLRSEQPY FKSDHGPIRS LPAKWENVSA QLSKNPKSPP PKRAECSSQK
         ********** ********** ******

mdec1    FAGSDLMGSP FLGPHPHQPP FCLPFYLIPP SATAYLPMLE KCWYPTSVPV LYPGLNTSAA
D8-1     ---------- ---------- ---------- ---------- ---------- ----------
D8-2     RKATSRAVI* ---------- ---------- ---------- ---------- ----------

mdec1    ALSSFMNPDK IPTPLLLPQR LPSPLAHSSL DSSALLQALK QIPPLNLETK D*
D8-1     ---------- ---------- ---------- ---------- ---------- --
D8-2     ---------- ---------- ---------- ---------- ---------- --
```

Figure S34. Predicted translation products of both alleles in the *Dec1* deletion cell.

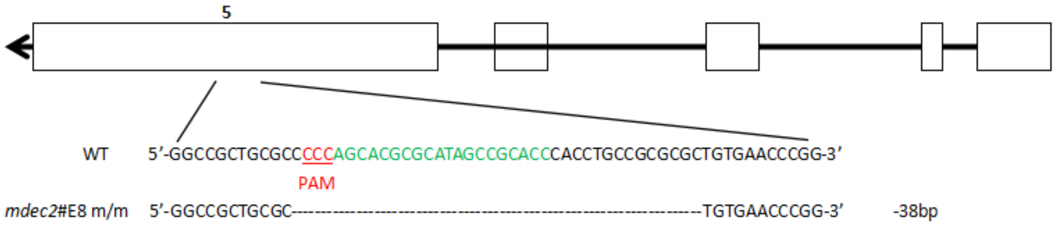

Figure S35. The sgRNA targeting the *Dec2* gene and the sequences of both alleles in the *Dec2* deletion cell strain.

```
mdec2       MDEGIPHLQE RQLLEHRDFI GLDYSSLYMC KPKRSLKRDD TKDTYKLPHR LIEKKRRDRI
E8-1/2      MDEGIPHLQE RQLLEHRDFI GLDYSSLYMC KPKRSLKRDD TKDTYKLPHR LIEKKRRDRI
            ********** ********** ********** ********** ********** **********
mdec2       NECIAQLKDL LPEHLKLTTL GHLEKAVVLE LTLKHLKALT ALTEQQHQKI IALQNGERSL
E8-1/2      NECIAQLKDL LPEHLKLTTL GHLEKAVVLE LTLKHLKALT ALTEQQHQKI IALQNGERSL
            ********** ********** ********** ********** ********** **********
mdec2       KSPVQADLDA FHSGFQTCAK EVLQYLARFE SWTPREPRCA QLVSHLHAVA TQLLTPQVPS
E8-1/2      KSPVQADLDA FHSGFQTCAK EVLQYLARFE SWTPREPRCA QLVSHLHAVA TQLLTPQVPS
            ********** ********** ********** ********** ********** **********
mdec2       GRGSGRAPCS AGAAAASGPE RVARCVPVIQ RTQPGTEPEH DTDTDSGYGG EAEQGRAAVK
E8-1/2      GRGSGRAPCS AGAAAASGPE RVARCVPVIQ RTQPGTEPEH DTDTDSGYGG EAEQGRAAVK
            ********** ********** ********** ********** ********** **********
mdec2       QEPPGDSSPA PKRPKLEARG ALLGPEPALL GSLVALGGGA PFAQPAAAPF CLPFYLLSPS
E8-1/2      QEPPGDSSPA PKRPKLEARG ALLGPEPALL GSLVALGGGA PFAQPAAAPF CLPFYLLSPS
            ********** ********** ********** ********** ********** **********
mdec2       AAAYVQPWLD KSGLDKYLYP AAAAPFPLLY PGIPAAAAAA AAAAFPCLSS VLSPPPEKAG
E8-1/2      AAAYVQPWLD KSGLDKYLYP AAAAPFPLLY PGIPAAAAAA AAAAFPCLSS VLSPPPEKAG
            ********** ********** ********** ********** ********** **********
mdec2       ATAGAPFLAH EVAPPGPLRP QHAHSRTHLP RAVNPESSQE DATQPAKDAP *
E8-1/2      ATAGAPFLAH EVAPPGPLRC EPGELSGRCH AAGQGRPL-- ---------- -
            ********** *********
```

Figure S36. Predicted translation products of both alleles in the *Dec2* deletion cell.

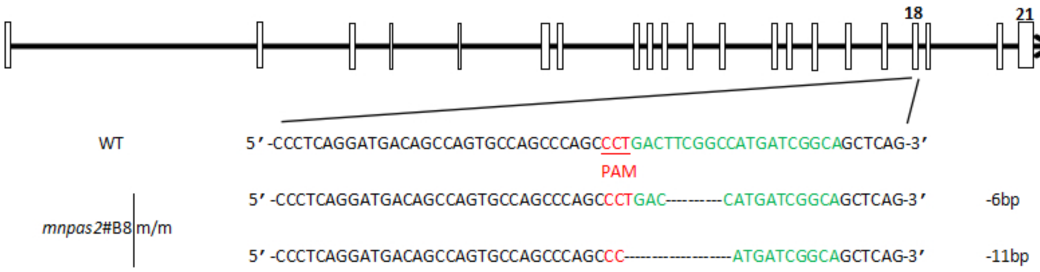

Figure S37. The sgRNA targeting the *Npas2* gene and the sequences of both alleles in the *Npas2* deletion cell strain.

```
mnpas2      MDEDEKDRAK RASRNKSEKK RRDQFNVLIK ELSSMLPGNT RKMDKTTVLE KVIGFLQKHN
B8-1        MDEDEKDRAK RASRNKSEKK RRDQFNVLIK ELSSMLPGNT RKMDKTTVLE KVIGFLQKHN
B8-2        MDEDEKDRAK RASRNKSEKK RRDQFNVLIK ELSSMLPGNT RKMDKTTVLE KVIGFLQKHN
            ********** ********** ********** ********** ********** **********

mnpas2      EVSAQTEICD IQQDWKPSFL SNEEFTQLML EALDGFVIVV TTDGSIIYVS DSITPLLGHL
B8-1        EVSAQTEICD IQQDWKPSFL SNEEFTQLML EALDGFVIVV TTDGSIIYVS DSITPLLGHL
B8-2        EVSAQTEICD IQQDWKPSFL SNEEFTQLML EALDGFVIVV TTDGSIIYVS DSITPLLGHL
            ********** ********** ********** ********** ********** **********

mnpas2      PADVMDQNLL NFLPEQEHSE VYKILSSHML VTDSPSPEFL KSDNDLEFYC HLLRGSLNPK
B8-1        PADVMDQNLL NFLPEQEHSE VYKILSSHML VTDSPSPEFL KSDNDLEFYC HLLRGSLNPK
B8-2        PADVMDQNLL NFLPEQEHSE VYKILSSHML VTDSPSPEFL KSDNDLEFYC HLLRGSLNPK
            ********** ********** ********** ********** ********** **********

mnpas2      EFPTYEYIKF VGNFRSYNNV PSPSCNGFDN TLSRPCHVPL GKDVCFIATV RLATPQFLKE
B8-1        EFPTYEYIKF VGNFRSYNNV PSPSCNGFDN TLSRPCHVPL GKDVCFIATV RLATPQFLKE
B8-2        EFPTYEYIKF VGNFRSYNNV PSPSCNGFDN TLSRPCHVPL GKDVCFIATV RLATPQFLKE
            ********** ********** ********** ********** ********** **********

mnpas2      MCVADEPLEE FTSRHSLEWK FLFLDHRAPP IIGYLPFEVL GTSGYDYYHI DDLELLARCH
B8-1        MCVADEPLEE FTSRHSLEWK FLFLDHRAPP IIGYLPFEVL GTSGYDYYHI DDLELLARCH
B8-2        MCVADEPLEE FTSRHSLEWK FLFLDHRAPP IIGYLPFEVL GTSGYDYYHI DDLELLARCH
            ********** ********** ********** ********** ********** **********

mnpas2      QHLMQFGKGK SCCYRFLTKG QQWIWLQTHY YITYHQWNSK PEFIVCTHSV VSYADVRVER
B8-1        QHLMQFGKGK SCCYRFLTKG QQWIWLQTHY YITYHQWNSK PEFIVCTHSV VSYADVRVER
B8-2        QHLMQFGKGK SCCYRFLTKG QQWIWLQTHY YITYHQWNSK PEFIVCTHSV VSYADVRVER
            ********** ********** ********** ********** ********** **********

mnpas2      RQELALEDPP TEAMHPSAVK EKDSSLEPPQ PFNALDMGAS GLPSSPSPSA SSRSSHKSSH
B8-1        RQELALEDPP TEAMHPSAVK EKDSSLEPPQ PFNALDMGAS GLPSSPSPSA SSRSSHKSSH
B8-2        RQELALEDPP TEAMHPSAVK EKDSSLEPPQ PFNALDMGAS GLPSSPSPSA SSRSSHKSSH
            ********** ********** ********** ********** ********** **********

mnpas2      TAMSEPTSTP TKLMAENSTT ALPRPATLPQ ELPVQGLSQA ATMPTALHSS ASCDLTKQLL
B8-1        TAMSEPTSTP TKLMAENSTT ALPRPATLPQ ELPVQGLSQA ATMPTALHSS ASCDLTKQLL
B8-2        TAMSEPTSTP TKLMAENSTT ALPRPATLPQ ELPVQGLSQA ATMPTALHSS ASCDLTKQLL
            ********** ********** ********** ********** ********** **********

mnpas2      LQSLPQTGLQ SPPAPVTQFS AQFSMFQTIK DQLEQRTRIL QANIRWQQEE LHKIQEQLCL
B8-1        LQSLPQTGLQ SPPAPVTQFS AQFSMFQTIK DQLEQRTRIL QANIRWQQEE LHKIQEQLCL
B8-2        LQSLPQTGLQ SPPAPVTQFS AQFSMFQTIK DQLEQRTRIL QANIRWQQEE LHKIQEQLCL
            ********** ********** ********** ********** ********** **********

mnpas2      VQDSNVQMFL QQPAVSLSFS STQRPAAQQQ LQQRPAAPSQ PQLVVNTPLQ GQITSTQVTN
B8-1        VQDSNVQMFL QQPAVSLSFS STQRPAAQQQ LQQRPAAPSQ PQLVVNTPLQ GQITSTQVTN
B8-2        VQDSNVQMFL QQPAVSLSFS STQRPAAQQQ LQQRPAAPSQ PQLVVNTPLQ GQITSTQVTN
            ********** ********** ********** ********** ********** **********

mnpas2      QHLLRESNVI SAQGPKPMRS SQLLPASGRS LSSLPSQFSS TASVLPPGLS LTTIAPTPQD
B8-1        QHLLRESNVI SAQGPKPMRS SQLLPASGRS LSSLPSQFSS TASVLPPGLS LTTIAPTPQD
B8-2        QHLLRESNVI SAQGPKPMRS SQLLPASGRS LSSLPSQFSS TASVLPPGLS LTTIAPTPQD
            ********** ********** ********** ********** ********** **********

mnpas2      DSQCQPSPDF GHDRQLRLLL SQPIQPMMPG SCDARQPSEV SRTGRQVKYA QSQVMFPSPD
B8-1        DSQCQPSPD- -HDRQLRLLL SQPIQPMMPG SCDARQPSEV SRTGRQVKYA QSQVMFPSPD
B8-2        DSQCQPSP*- ---------- ---------- ---------- ---------- ----------
            ********

mnpas2      SHPTNSSAST PVLLMGQAVL HPSFPASRPS PLQPAQAQQQ PPPYLQAPTS LHSEQPDSLL
B8-1        SHPTNSSAST PVLLMGQAVL HPSFPASRPS PLQPAQAQQQ PPPYLQAPTS LHSEQPDSLL
B8-2        ---------- ---------- ---------- ---------- ---------- ----------

mnpas2      LSTFSQQPGT LGYAATQSTP PQPPRPSRRV SRLSES*
B8-1        LSTFSQQPGT LGYAATQSTP PQPPRPSRRV SRLSES*
B8-2        ---------- ---------- ---------- -------
```

Figure S38. Predicted translation products of both alleles in the *Npas2* deletion cell.

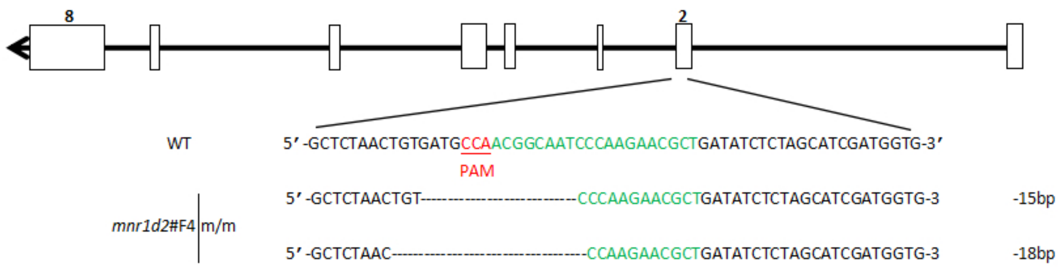

Figure S39. The sgRNA targeting the *Nr1d2* gene and the sequences of both alleles in the *Nr1d2* deletion cell strain.

```
mnr1d2      MELNAGGVIA YISSSSSASS PASCHSEGSE NSFQSSSSSV PSSPNSSNCD ANGNPKNADI
F4-1        MELNAGGVIA YISSSSSASS PASCHSEGSE NSFQSSSSSV PSSPNSSNC- ----PKNADI
F4-2        MELNAGGVIA YISSSSSASS PASCHSEGSE NSFQSSSSSV PSSPNSSNPR TLISLASMVF
            ********** ********** ********** ********** ********
mnr1d2      SSIDGVLKSD RTDCPVKTGK TSAPGMTKSH SGMTKFSGMV LLCKVCGDVA SGFHYGVHAC
F4-1        SSIDGVLKSD RTDCPVKTGK TSAPGMTKSH SGMTKFSGMV LLCKVCGDVA SGFHYGVHAC
F4-2        *--------- ---------- ---------- ---------- ---------- ----------

mnr1d2      EGCKGFFRRS IQQNIQYKKC LKNENCSIMR MNRNRCQQCR FKKCLSVGMS RDAVRFGRIP
F4-1        EGCKGFFRRS IQQNIQYKKC LKNENCSIMR MNRNRCQQCR FKKCLSVGMS RDAVRFGRIP
F4-2        ---------- ---------- ---------- ---------- ---------- ----------

mnr1d2      KREKQRMLIE MQSAMKTMMN TQFSGHLQND TLAEQHDQSA LPAQEQLRPK SQLEQENIKN
F4-1        KREKQRMLIE MQSAMKTMMN TQFSGHLQND TLAEQHDQSA LPAQEQLRPK SQLEQENIKN
F4-2        ---------- ---------- ---------- ---------- ---------- ----------

mnr1d2      TPSDFAKEEV IGMVTRAHKD TFLYNQEHRE NSSESMPPQR GERIPRNMEQ YNLNQDHRGS
F4-1        TPSDFAKEEV IGMVTRAHKD TFLYNQEHRE NSSESMPPQR GERIPRNMEQ YNLNQDHRGS
F4-2        ---------- ---------- ---------- ---------- ---------- ----------

mnr1d2      GIHNHFPCSE RQQHLSGQYK GRNIMHYPNG HAVCIANGHC MNFSSAYTQR VCDRIPVGGC
F4-1        GIHNHFPCSE RQQHLSGQYK GRNIMHYPNG HAVCIANGHC MNFSSAYTQR VCDRIPVGGC
F4-2        ---------- ---------- ---------- ---------- ---------- ----------

mnr1d2      SQTENRNSYL CNTGGRMHLV CPMSKSPYVD PQKSGHEIWE EFSMSFTPAV KEVVEFAKRI
F4-1        SQTENRNSYL CNTGGRMHLV CPMSKSPYVD PQKSGHEIWE EFSMSFTPAV KEVVEFAKRI
F4-2        ---------- ---------- ---------- ---------- ---------- ----------

mnr1d2      PGFRDLSQHD QVNLLKAGTF EVLMVRFASL FDAKERTVTF LSGKKYSVDD LHSMGAGDLL
F4-1        PGFRDLSQHD QVNLLKAGTF EVLMVRFASL FDAKERTVTF LSGKKYSVDD LHSMGAGDLL
F4-2        ---------- ---------- ---------- ---------- ---------- ----------

mnr1d2      SSMFEFSEKL NALQLSDEEM SLFTAVVLVS ADRSGIENVN SVEALQETLI RALRTLIMKN
F4-1        SSMFEFSEKL NALQLSDEEM SLFTAVVLVS ADRSGIENVN SVEALQETLI RALRTLIMKN
F4-2        ---------- ---------- ---------- ---------- ---------- ----------

mnr1d2      HPNEASIFTK LLLKLPDLRS LNNMHSEELL AFKVHP*
F4-1        HPNEASIFTK LLLKLPDLRS LNNMHSEELL AFKVHP*
F4-2        ---------- ---------- ---------- -------
```

Figure S40. Predicted translation products of both alleles in the *Nr1d2* deletion cell.

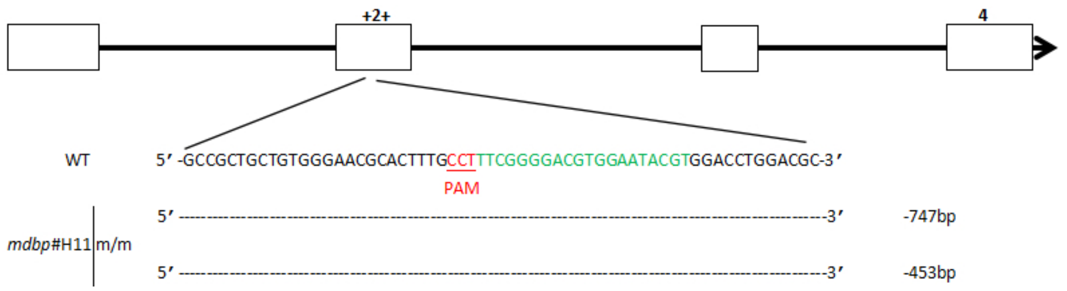

Figure S41. The sgRNA targeting the *Dbp* gene and the sequences of both alleles in the *Dbp* deletion cell strain.

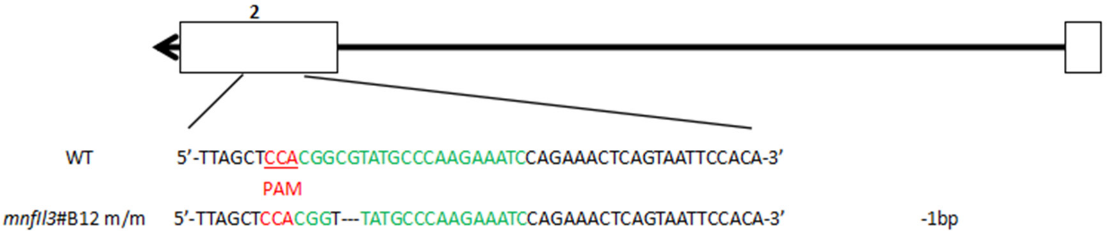

Figure S42. The sgRNA targeting the *Nfil3* gene and the sequences of both alleles in the *Nfil3* deletion cell strain.

```
mnfil3     MQLRKMQTIK KEPAPLDPTS SSDKMLLLNS ALAEVAEDLA SGEDLLLNEG SMGKNKSSAC
B12-1/2    MQLRKMQTIK KEPAPLDPTS SSDKMLLLNS ALAEVAEDLA SGEDLLLNEG SMGKNKSSAC
           ********** ********** ********** ********** ********** **********

mnfil3     RRKREFIPDE KKDAMYWEKR RKNNEAAKRS REKRRLNDLV LENKLIALGE ENATLKAELL
B12-1/2    RRKREFIPDE KKDAMYWEKR RKNNEAAKRS REKRRLNDLV LENKLIALGE ENATLKAELL
           ********** ********** ********** ********** ********** **********

mnfil3     SLKLKFGLIS STAYAQEIQK LSNSTAVYFQ DYQTSKAAVS SFVDEHEPAM VAGSCISVIK
B12-1/2    SLKLKFGLIS STVMPKKSRN SVIPQLSTFR TTRHPRLP*- ---------- ----------
           ********** **

mnfil3     HSPQSSLSDV SEVSSVEHTQ ESPAQGGCRS PENKFPVIKQ EPVELESFAR EAREERGTYS
B12-1/2    ---------- ---------- ---------- ---------- ---------- ----------

mnfil3     TSIYQSYMGS SFSTYSHSPP LLQVHGSTSN SPRTSEADEG VVGKSSDGED EQQVPKGPIH
B12-1/2    ---------- ---------- ---------- ---------- ---------- ----------

mnfil3     SPVELQRVHA TVVKVPEVNP SALPHKLRIK AKAMQVKVEA LDSEFEGMQK LSSPADAIAK
B12-1/2    ---------- ---------- ---------- ---------- ---------- ----------

mnfil3     RHFDLEKHGT SGMAHSSLPP FSVQVTNIQD WSLKSEHWHH KELSSKTQSS FKTGVVEVKD
B12-1/2    ---------- ---------- ---------- ---------- ---------- ----------

mnfil3     GGYKVSEAEN LYLKQGIANL SAEVVSLKRF IATQPISASD SR*
B12-1/2    ---------- ---------- ---------- ---------- ---
```

Figure S43. Predicted translation products of both alleles in the *Nfil3* deletion cell.

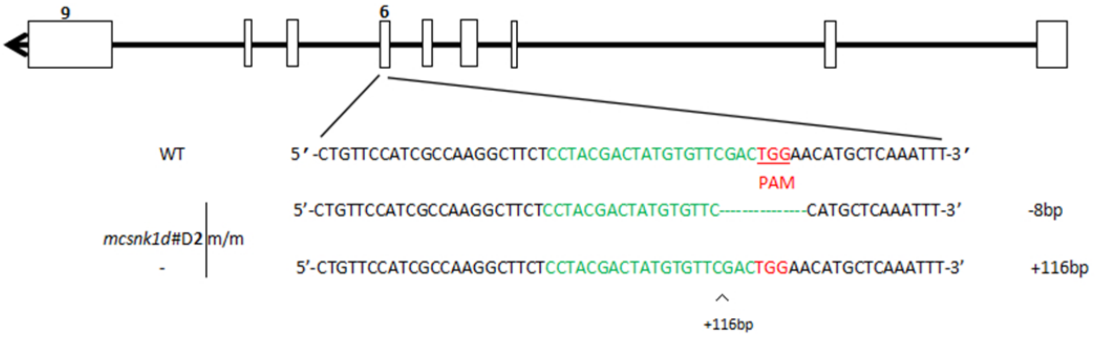

Figure S44. The sgRNA targeting the *Csnk1d* gene and the sequences of both alleles in the *Csnk1d* deletion cell strain (Knocking-out of CK1d was reported in Guo et al., JBR, 2019).

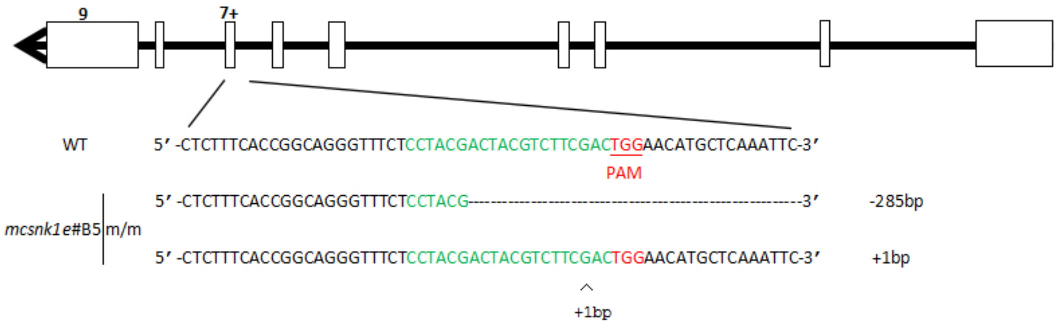

Figure S45. The sgRNA targeting the *Csnk1e* gene and the sequences of both alleles in the *Csnk1e* deletion cell strain (Knocking-out of CK1e was reported in Guo et al., JBR, 2019).

Note: No translation predictions on *Dbp*-H11, *Per2*-A2, and *Rorb*-B3 cell lines, due to the impairments of splicing signals in the targeted exons by mutated nucleotides.

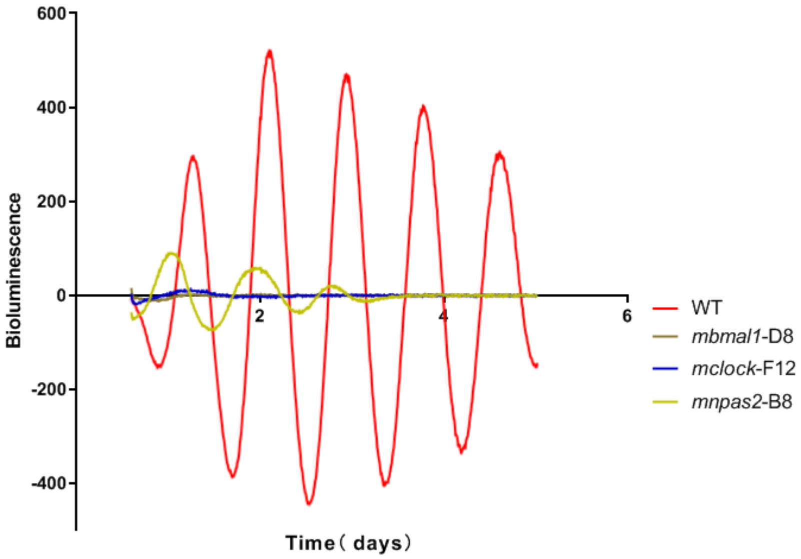

Figure S46. The bioluminescence of the wild-type, Bmal1-D8, Clock-F12, and Npas2-B8 cells. These genes belong to the core positive regulator group.

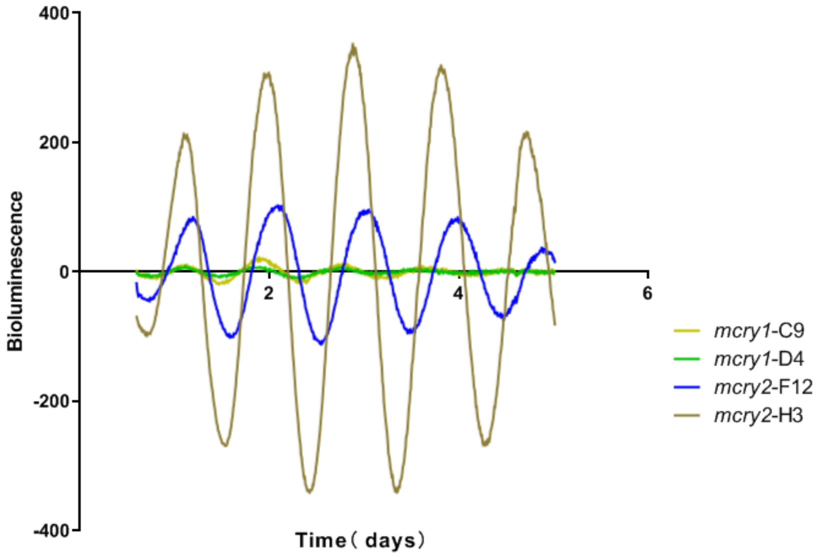

Figure S47. The bioluminescence of the Cry1 and Cry2 knock-out cells.

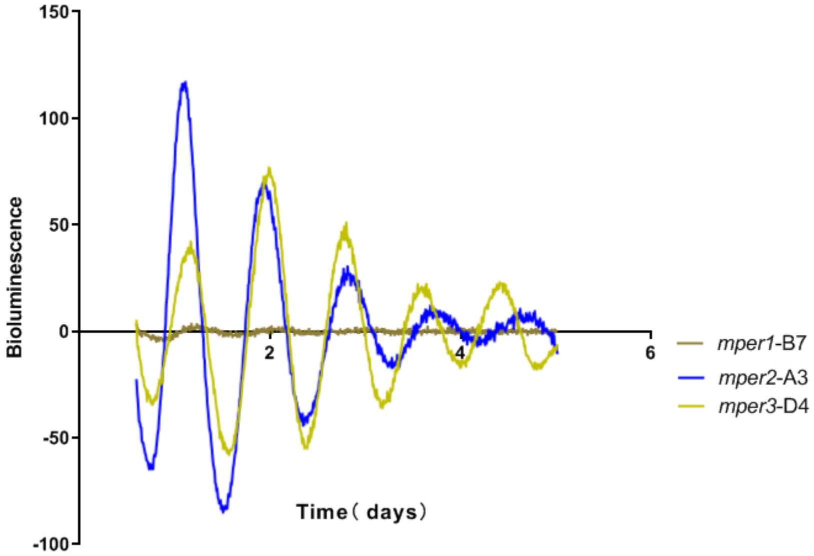

Figure S48. The bioluminescence of the Per1, Per2, and Per3 knock-out cells.

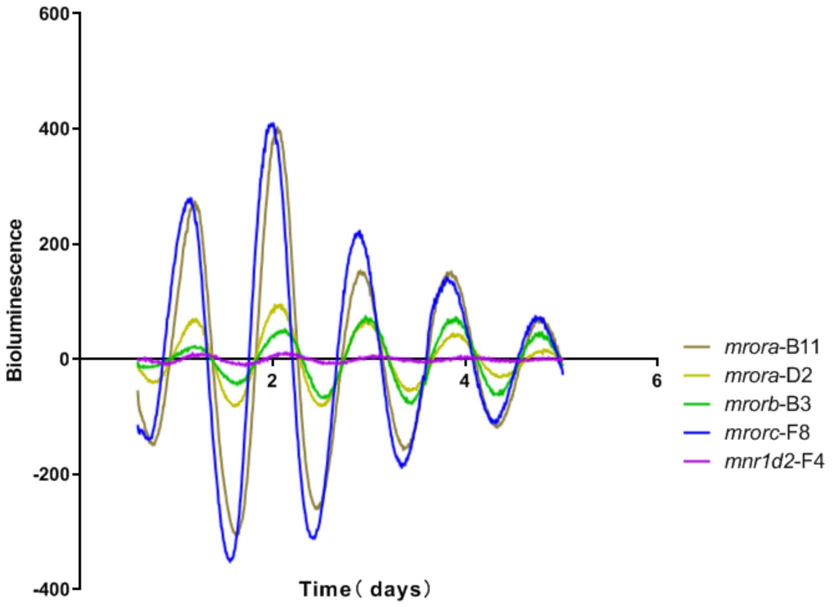

Figure S49. The bioluminescence of the Rora-B11, Rora-D2, Rorb-B3, Rorc-F8, Nr1d2-F4 cells. These genes represent regulators for the Bmal1 gene.

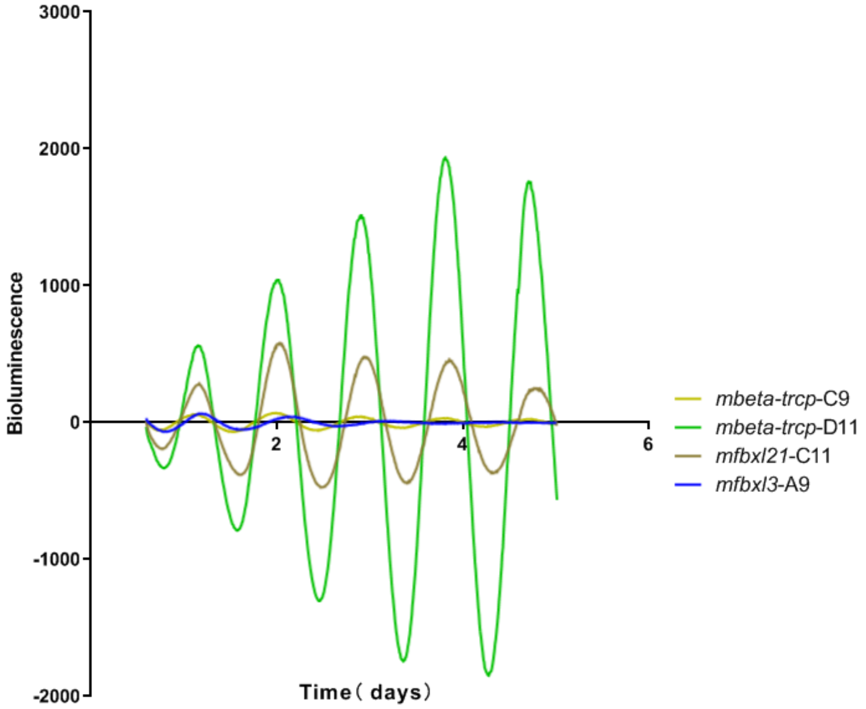

Figure S50. The bioluminescence of the Beta-trcp-C9, Beta-trcp-D11, Fbxl21-C11, Fbxl3-A9 cells. These genes represent E3 ligases.

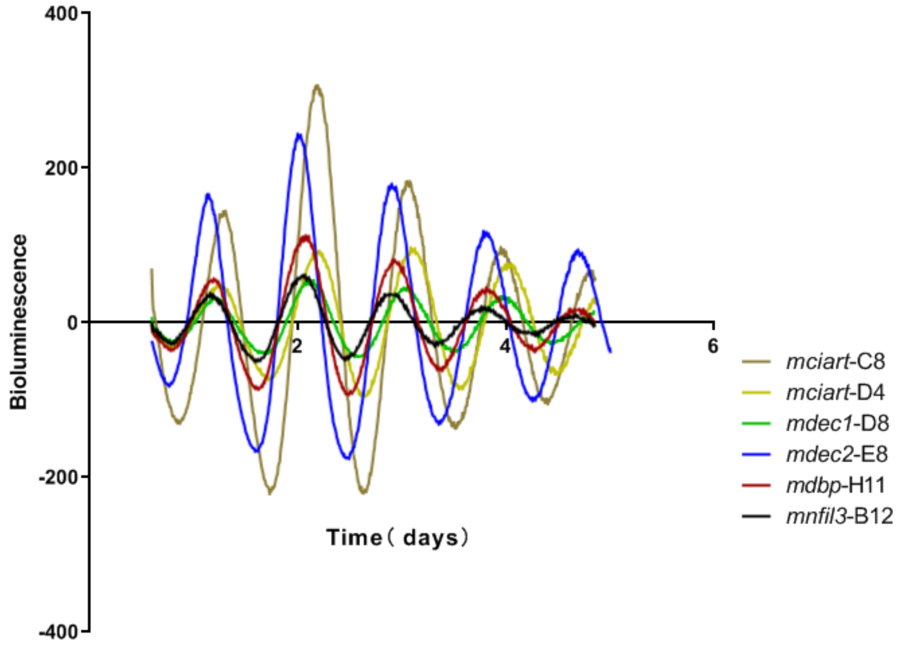

Figure S51. The bioluminescence of the Ciart-C8, Ciart-D4, Dec1-D8, Dec2-E8, Dbp-H11, Nfil3-B12 cells. These represent auxiliary regulators.

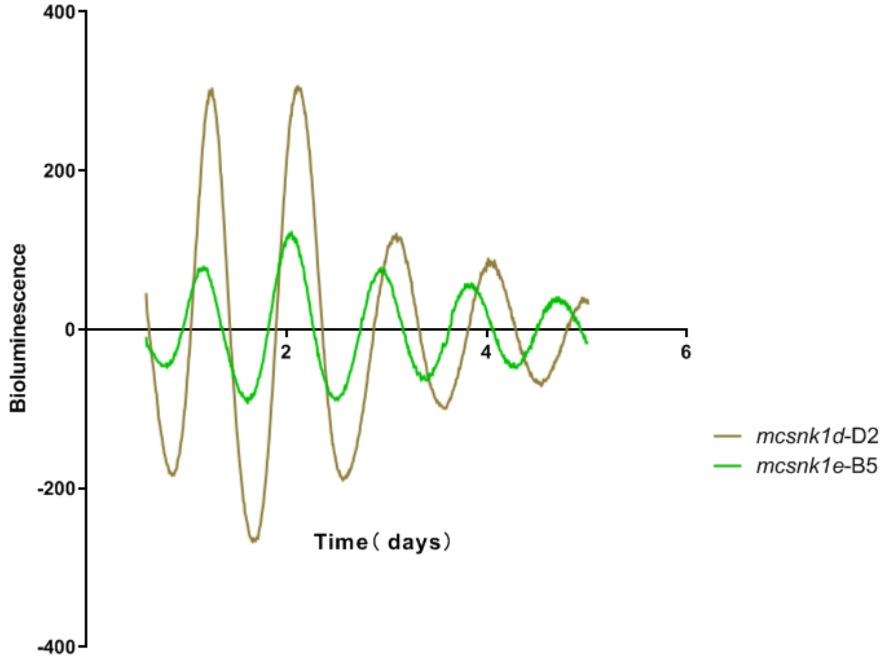

Figure S52. The bioluminescence of the casein kinase genes, Csnk1d-D2, Csnk1e-B5 cells.

Table S1. The sequence information of sgRNAs used in this study.

Target gene	sequence	Loction-Exon	Score
Bmal1	ACCCGTATTTCCCCGTTCGC**TGG**	14/21	98
Clock	TCCATCTTTCTCGCGTTACC**AGG**	6/23	94
Npas2	TGCCGATCATGGCCGAAGTC**AGG**	18/21	95
Cry1	TTCGCCGGCTCTTCCAACGT**GGG**	1/13	95
Cry2	ACGGTCCCCGCGCAATCGAT**GGG**	1/12	99
Per1	TCGCTTCTGTGCTCGGAACG**GGG**	11/23	93
Per2	TCGATTCCGCACCCGCAACG**GGG**	11/23	99
Per3	GCCGCACTGAGCGTGGCGTC**TGG**	15/21	96
Rora	ACGCGATTTCGTCTTCGGTC**AGG**	9/11	98
Rorb	ACCGGCGGCACATACGCCAA**CGG**	4/10	97
Rorc	GTTGCGGCTGGTTCGGTCAA**TGG**	3/10	97
Nr1d1	GTTGCGATTGATGCGAACGA**TGG**	4/8	99
Nr1d2	AGCGTTCTTGGGATTGCCGT**TGG**	2/8	91
Dbp	ACGTATTCCACGTCCCCGAA**AGG**	2/4	95
Nfil3	GATTTCTTGGGCATACGCCG**TGG**	2/2	98
Ciart	CAGCCCCGGGGTCACCGTGG**TGG**	5/5	79
Dec1	CATGGACGCAGGTTCGCCGT**GGG**	5/5	92
Dec2	GGTGCGGCTATGCGCGTGCT**GGG**	5/5	97
Beta-trcp	TGGTACCGCGTGACGTCGGA**CGG**	6/15	98
Fbxl21	GCCCGGGCTCGATCTATTAA**AGG**	4/7	97
Fbxl3	TCGGACTTATCTCGACTGCC**CGG**	3/5	95
Myc	GCTGTACGGAGTCGTAGTCG**AGG**	2/3	98
Timeless	TACTCGAGCCCGTGAACGTT**TGG**	23/29	98
Csnk1d	CCTACGACTATGTGTTCGAC**TGG**	6/9	95
Csnk1e	CCTACGACTACGTCTTCGAC**TGG**	7/9	98

Note：Location-Exon means the number of sgRNA's exon /total exon

Table S2. The sequence information of primers to amplify targeted sites

GeneName	Forward primer (5'to 3')	Reverse primer (5'to 3')	Product length
Bmal1-C	AGGTGAGACCGAATCCCACT	TAGAAAAGCCAGCTGACCACC	664bp
Clock-C	CTGTCTCCCCAGGCTTACG	TGACATATTTGAGCCAGGCGTG	569bp
Npas2-C	TGCCATCCCAGTTTAGCAGT	GGGTAGTGTACTCAAGCCGAG	552bp
Cry1-C	CTCCACACTATGAGTTACCTAG ACAC	GAGCTCGTGTCCGTTCGTGGAG	983bp
Cry2-C	CCCACAGCACACCCCTAAAA	GAGGCGATCCAGACTCCTTG	594bp
Per1-C	GATCTTCACCACCCGACACA	CCAGCGTGGAGACACAGTT	585bp
Per2-C	ATCTCTATCCCACAGACCCCC	GTTGGACCCACTGGGAATGC	613bp
Per3-C	GGCCTTAGCTGTCCGTGTAA	TCACCTGGTGCAGCCAATAA	518bp
Rora-C	CAGCTGACAGTGGTGGCTAA	CCGCTGAGCCATCTTTTCAC	614bp
Rorb-C	AGGGCAAGGGGCTAGTGATA	GAGCATGGTGCCTAGAGAGAG	1050bp
Rorc-C	ATCTGGGATCCACTACGGGG	CCCCCACATTCTCACCATCTC	593bp
Nr1d1-C	GGGGCGAGAAGGAGATATGG	AGCTCCTATCCAGTCCAGTCC	512bp
Nr1d2-C	ATGCCAGGAGCACTGGTTTT	CTGGTGTGAGCCAGGAATGT	547bp
Beta-trcp-C	TCAGGCATGTGCTGCTCTTT	TGTCTCTCAGGCTTTCTGCAA	562bp
Fbxl3-C	CGACCGGATCAGAAAGCTCA	TGGACACCCTTGCTTCACTC	1084bp
Fbxl21-C	GCACCAATCAATGCGTGGAA	AACAGCCCAGTGAGAAACTG	726bp
Ciart-C	GGACAGGCGGACATAGGATG	ACACACCTGTTGCACAAACG	555bp
Dbp-C	GGCCCCTAACCCTATCCTTC	CTGCTCGCTCCCATTTTTGG	991bp
Nfil3-C	ATGGAGGTGGAATACGTGCC	TTGTACCAGTACCCGCTGAC	981bp
Dec1-C	CTCGTCACTCATCTCCATCGTG	GATCTGCTTCAAAGCCTGGAGC	696bp
Dec2-C	CGTACAGCCCTGGCTAGACAAG	CTGTTCCAGGCTTGGGTCGAG	715bp
Csnk1d-C	ATCCAGAAGGAGCTGACTCTGA GC	TGAGCTGTACCTCTACTTAGATGC TG	800bp
Csnk1e-C	CTCAGAAAGGGCCACCACTT	TCATCCCGCCCCTCTTAGAT	651bp
Myc-C	GTATTCCCTACAGTCGCCTCC	CGACCGCAACATAGGATGGA	585bp
Timeless-C	AGGTAAAGGCATGGGCTCTG	CTACAGTGCTTTGTGGGGTGA	527bp

Table S3. The most possible off-target sites for each individual gene in this study

Target gene	sequence	Chromosome	Site	Score
Bmal1	ACCCTTATTTTCCCGTTCCCAAG	17	-91174644	0.6
Clock	TCCTTCCTTCTCCCGTTACCAAG	13	42768150	0.7
Npas2	TGCTGATCATCGCCAAAGTCTAG	15	-8659777	0.4
Cry1	TCCACCGGTCCTTCCAACGTCAG	10	-41714430	0.8
Cry2	CCTGTCCCCGTGCAATCGACTAG	11	111524673	0.4
Per1	TGCGTACTGTGCTCGGAACGCGG	11	51817733	0.8
Per2	TCCCTTCCGCAGCCGCGACGGAG	6	-100094135	0.3
Per3	GCAACACTGAGCGAGGCGTCGGG	13	12487247	0.4
Rora	AAGTGATTTCTTCTTCAGTCAAG	8	-112844682	0.3
Rorb	ACCGGCGGCCCAGACGCCAGCAG	6	-71781796	0.4
Rorc	GTGGAGGGTGGTTCGGTCACAGG	8	118850237	0.7
Nr1d2	AGGCTTCCTGTGATTGCCGTCAG	7	-119338549	0.8
Dbp	TCTTATTCCACCTCCCCGAATAG	9	-66088878	1.4
Nfil3	GATTCCTTGAGTATACGCCAGGG	4	86171320	0.3
Ciart	CTGTCCCGGGGTCACCATGGCAG	2	-131948868	1.3
Dec1	CCGGGAGGCAGGGTCGCCGTGAG	10	-61461408	0.4
Dec2	GGAGCAGACATGCGCGTGCTGGG	11	68844738	0.5
Beta-trcp	CGGGACAGCGTGACGTCGCAAGG	4	-151671218	0.4
Fbxl21	CCCAGGGCCCTATCTATTAAAGG	2	-14610824	0.5
Fbxl3	TGGGACTTGTCTCGACTGCATAG	6	58683716	0.9
Csnk1d	GGTTGGGGAAGCCTTGGCGACAG	7	51888871	1.7
Csnk1e	CCTACGACTATGTGTTCGACTGG	11	-120832891	0.5

Table S4. Primers to amplify the most possible off-target site for individual genes

GeneName	Forward primer (5'to 3')	Reverse primer (5'to 3')	Product length
Bmal1-O	ACGATGAGGAGGTAGTGA	TGTAAGTGGAAATGGGTA	618bp
Clock-O	AGTGCGGTTCTGCTATT	TTTCTGTCAACGCTTCA	488bp
Npas2-O	CATTAGAAGGTAGGAGCAT	CAAGATTGAACGGGTGT	601bp
Cry1-O	GTCATCCTCCTGCCACT	GTCCCATCAACTAATCCTT	494bp
Cry2-O	GTGTTACAGGCTTCCC	CTCAGCAGGTGTTTCC	199bp
Per1-O	CCCCATAAGTGTTCCGTAGA	CCTCAGCCCATCCTGTAGTC	499bp
Per2-O	CTTTGCTCACTCCCTCTGG	TCTGGCGATTTGTCTTCC	395bp
Per3-O	CTGCGTTCAGCGAGTG	TCTAAGTTCCGCCTATTCA	545bp
Rora-O	GAGAACGGAAGGTGAG	CAGCATCCTGGAGACA	548bp
Rorb-O	ACTGCCAACGGACAACG	CTCGGCTACGGAAACAAA	445bp
Rorc-O	GTTCTTGGACGCTCAC	GCCTCCTGGATGTTG	223bp
Nr1d1-O	GGTCAAGGGAAATAAAG	GGGTCCTAACGCAGT	555bp
Nr1d2-O	AAGGGTCCTGCTCCA	GATTCTTAGGCATTCACG	529bp
Beta-trcp-O	GGGCTCTAAACTTGGGACGAA	GCGGTTGAGGGCTGGATT	162bp
Fbxl3-O	AAACCCGAAGTCAGTAG	GATGGCAATCCAAGTAG	416bp
Fbxl21-O	TTTATCTCCTGCTTCTACT	CTTCTGCCTCAACCC	936bp
Ciart-O	GCAAGGGTTCAAGGC	AGGCAAATGGTGGTTAG	500bp
Dbp-O	TGGCAGCAGCACCTAA	CACCACCATCTCAGACACC	776bp
Nfil3-O	AAACAAGTGGGTAAGTGC	GTTGCCCGATACAGATT	503bp
Dec1-O	CACCATTGTTCCCTT	CTGCTCCTCAGTGTTAG	550bp
Dec2-O	TCATCTTTCTTGGCTCTTC	TCCCGTGCTGACTTCTT	255bp
Csnk1d-O	GCACTTGTAGGGACTTGG	CTGCGGTGATAGGAGGT	469bp
Csnk1e-O	TTGGCTTTCACAACTCTA	TTTCTGCCGTTCCTTA	269bp
Myc-O	TTGCTGAACGGACAAATAC	CGGGACCAATGGAGACA	1039bp
Timeless-O	TCCCTGCTATTGCTGC	ATGGGTTTGGTCTTTCC	308bp
